# Cost-effectiveness of MLC601 in post-stroke functional recovery compared with placebo - the CHIMES & CHIMES-E studies

**DOI:** 10.1186/s12913-024-11618-4

**Published:** 2024-09-27

**Authors:** Christopher Li Hsian Chen, Jia Hui Chai, Yogesh Mahadev Pokharkar, Narayanaswamy Venketasubramanian

**Affiliations:** 1 Memory Aging and Cognition Centre, Department of Pharmacology, Yong Loo Lin School of Medicine, National University of Singapore, Blk MD3, 16 Medical Drive, #04-01, Singapore, 117600 Singapore; 2 Singapore Clinical Research Institute, Consortium for Clinical Research and Innovation Singapore, 23 Rochester Park, Singapore, 139234 Singapore; 3 Raffles Neuroscience Centre, Raffles Hospital, 585 North Bridge Road, #09-00, Singapore, 188770 Singapore

**Keywords:** Cost, Cost-effectiveness, Cost-utility, Functional recovery, MLC601, MLC901, NeuroAiD, Stroke

## Abstract

**Background:**

Despite progress in stroke therapy (e.g., revascularisation interventions by thrombolysis and/or thrombectomy, organised stroke care), many stroke survivors will have impairment of neurological function. We aimed to compare the cost-effectiveness of an oral natural formulation, MLC601, versus placebo in functional recovery among subjects receiving standard of care after an ischemic stroke of intermediate severity assessed with NIH Stroke Scale at baseline (b-NIHSS 8–14).

**Methods:**

A Markov cohort model with a 2-year time horizon was developed to simulate patients from a published randomised placebo-controlled clinical trial of MLC601 in their post-stroke functional recovery assessed by modified Rankin Score (mRS), from a health system perspective. Transition probabilities were derived from a multi-centre clinical trial in South East Asia. As cost and utility data were not collected in the trial, therefore we extracted them from the published literature. The main outcomes were incremental cost, incremental quality-adjusted life-year (QALY) gained, and incremental cost-effectiveness ratio (ICER). Besides base-case and sensitivity analyses, we performed subgroup analyses to explore the heterogeneity of patients with poor-prognosis factors (b-NIHSS 10–14, stroke onset to treatment time > 48 h, rehabilitation during first 3 month). All costs are expressed in 2022 Euro and USD, with an annual discount rate of 3% applied to costs and QALYs.

**Results:**

Base-case analysis showed that MLC601 was cost-effective compared with placebo, with €5,080 saved and 0.45 QALY gained, resulting in an ICER of -€11,352.50 per QALY gained. Similarly, results from subgroup analyses indicated that the use of MLC601 was a dominant strategy in all subgroups with poor-prognosis factors. Sensitivity analyses revealed the results were robust.

**Conclusion:**

Compared with placebo on top of standard stroke care, MLC601 was cost-effective in post-stroke functional recovery over two years. Due to the lack of cost and utility data from the study population, the results might not be generalizable to other settings. Further studies with country-specific data are needed to confirm the results of this study.

**Trial registration:**

URL http://www.clinicaltrials.gov. Unique identifier NCT00554723 November 7, 2007.

**Supplementary Information:**

The online version contains supplementary material available at 10.1186/s12913-024-11618-4.

## Background

Stroke is a major healthcare burden [1] and causes substantial economic losses worldwide [2]. The high cost of stroke management is related to the lengthy hospital care required, and even after discharge, the costs of extended care for risk management and prolonged rehabilitation. Despite the efforts made by health authorities in many countries to raise awareness about prevention of risk factors [3], or reduction of delays in accessing revascularisation interventions (thrombolysis and/or thrombectomy) [4, 5] and organised stroke care [6], many stroke survivors will have residual impairment of neurological function [7].

In these subjects with post-stroke functional impairment, the brain may spontaneously initiate self-repair processes, but these will most often be insufficient to achieve satisfactory functional recovery [8]. MLC601 (NeuroAiD), a natural herbal oral multi-ingredient formulation, has demonstrated neurorepair properties in animal and cellular models of cerebral ischemia [9]. These properties have been confirmed in a clinical study with an extended follow-up over 2 years [10, 11]. In a recent research study, we showed that MLC601 reduced the time to achieve functional recovery assessed by modified Rankin Score (mRS) compared to placebo [12]. Hence, we now analyse how these time savings translate into healthcare resource savings.

## Methods

### Objective

The analysis presented here aims to evaluate the potential cost-effectiveness of MLC601 in post-stroke functional recovery (i.e., mRS score 0 or 1) as compared to placebo in patients who received a 3-month oral course of MLC601 after an acute ischemic stroke (AIS).

### Study design

Similar data as those utilised for a time-saving analysis [12] extracted from a large randomised placebo-controlled clinical trial, the CHInese Medicine Neuroaid Efficacy on Stroke recovery (CHIMES) and its extension (CHIMES-E) double-blind follow-up study, were used for this analysis [10, 11]. Conducted in South East Asia (SEA), this international, multicentre, randomised, double-blind study compared a 3-month course of MLC601 to placebo in post-stroke recovery assessed with mRS at discharge or Day 10, and months 1, 3, 6, 12, 18 and 24. MLC601 is a multi-ingredient formulation derived from traditional Chinese medicine by Moleac Pte. (MLC) and marketed under the trade name NeuroAiD. The study treatment was administered three times a day in capsules containing extracts from raw herbal and non-herbal components [10], given on top of standard stroke care including control of vascular risk factors and rehabilitation as prescribed by investigators. The two studies were approved by the institutional review board of every study centre, and written informed consent collected from all subjects or legal representatives.

### Study population

In this exploratory analysis, the study population includes a subset of subjects randomised in the CHIMES study after an AIS of moderate severity with baseline NIH Stroke Scale score from 8 to 14 (b-NIHSS 8–14). Stroke severity was determined at baseline using the 15-item NIHSS, assessed within 72 h after stroke onset. We only considered subjects with b-NIHSS 8–14 as an analysis of all subjects (b-NIHSS 6–14) showed that subgroup with b-NIHSS 6–7 had limited symptoms and a strong tendency to recover spontaneously. At month 3, 68% of these subjects recovered in both treatment groups, while only 32% had recovered in the rest of the CHIMES population (b-NIHSS 8–14).

### Economic evaluation

#### Model structure

**Fig. 1:**
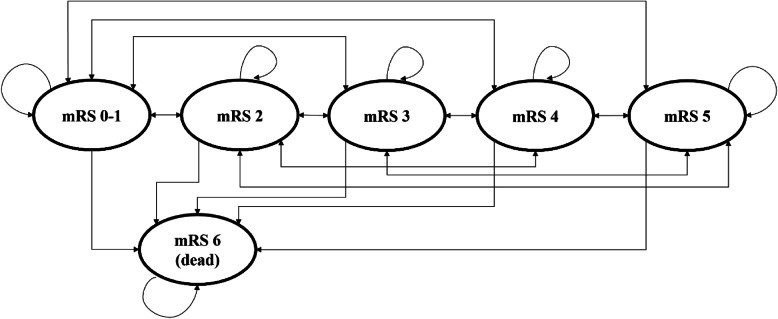
Markov model imitates the movement of stroke recovery, defined by modified Rankin Scale (mRS). A Markov model was developed to model the transition states of functional recovery of stroke measured by mRS, a commonly used scale to assess post-stroke functional disability: mRS 0 to 1 scores defined as functional recovery, mRS 2 to mild disability, mRS 3 to moderate disability, mRS 4 to moderately severe disability, mRS 5 to severe disability and mRS 6 to death. The arrows attached to each health state illustrate the movements of patients from one health state to another at every cycle. Each health state has movements outwards (to other health states) and inwards (pointing back to its own health state), except for health state mRS 6, defined as dead which is an absorbing health state because dead patients always remain in dead state

A Markov model, as illustrated in Fig. 1, was developed to model the transition states of functional recovery of stroke measured by mRS, a commonly used scale to assess post-stroke functional disability and neurorehabilitation trajectory in cost-effectiveness studies [13, 14]. The mRS scores range from 0 to 6, the definition of the scores being described in Fig. 1. In this analysis, we combined both mRS 0 and 1 scores to define functional recovery, with six health states in the Markov model: mRS 0–1, mRS 2, mRS 3, mRS 4, mRS 5, and mRS 6. The arrows attached to each health state illustrate the movements of patients from one health state to another at every cycle. Each health state has movements outwards (to other health states) and inwards (pointing back to its own health state), except for health state mRS 6 defined as death.

**Fig. 2:**
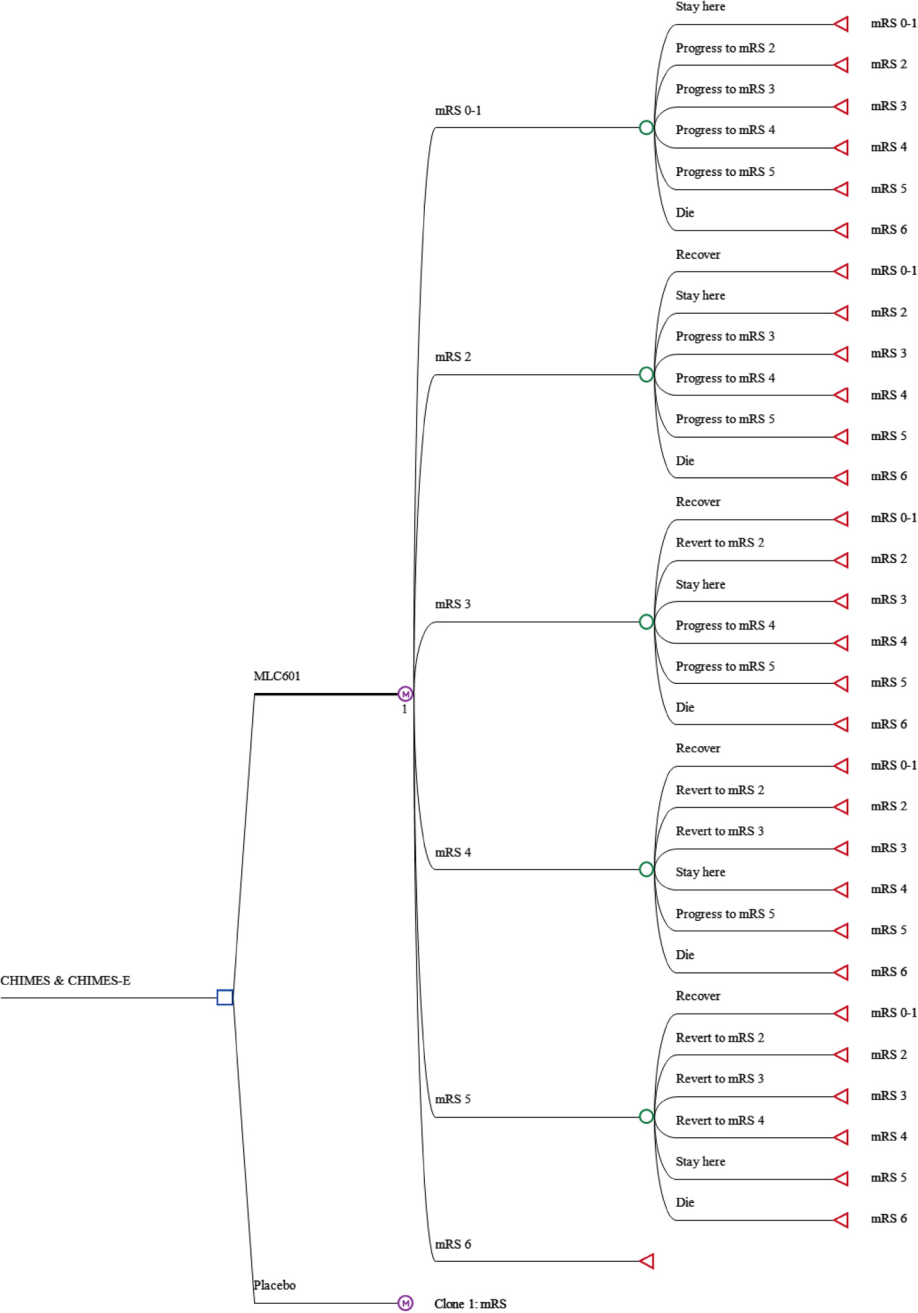
Decision-analytical model to estimate the cost and utility of MLC601 and placebo. A decision-analytical model was built to estimate and compare cost, effectiveness, and incremental cost-effectiveness ratio (ICER) between MLC601 and placebo groups. The decision-analytical model starts from left with a decision node with two branches indicating the two treatment strategy arms: MLC601 and placebo. The first branch is the treatment arm MLC601 it utilized the Markov model defined earlier (Fig. 1) to model the post-stroke recovery of patients at a cohort level. Patients transitioned between states in the Markov model every cycle until they reached the time horizon set for the model. Similarly, in the second branch for placebo arm, the identical Markov model structure was used to model the functional recovery of stroke patients

Decision-analytical model in Fig. 2 was built to estimate and compare cost, effectiveness, and incremental cost-effectiveness ratio (ICER) between MLC601 and placebo arms. Placebo was the comparator whilst MLC601 was the intervention group, according to the design of the CHIMES and CHIMES-E study [10, 11]. The decision-analytical model starts from left with a decision node with two branches indicating the two treatment strategy arms: MLC601 and placebo. The first branch of the treatment arm is MLC601; it utilised the Markov model defined earlier (Fig. 1) to model the post-stroke recovery of patients at a cohort level. Patients transitioned between states in the Markov model every cycle until they reached the time horizon set for the model. Similarly, in the second branch for placebo arm, the identical Markov model structure was used to model the functional recovery of stroke patients.

The time horizon of the model was 2 years to mirror the study duration of CHIMES-E, and an annual discount rate of 3% was applied to both costs and quality-adjusted life-years (QALYs). We adopted a health system perspective to reflect the impact of implementing MLC601 to stroke patients in their recovery at the health system level and to inform decision-makers for possible reimbursement.

#### Transition probabilities

Time-dependent transition probabilities were calculated from the mRS data from CHIMES and CHIMES-E to feed into the Markov model. The CHIMES and CHIMES-E study collected mRS data at day 10 or discharge, month 1, month 3, month 6 and every 6 months up to month 24. We defined the cycle length of the model as 3 months because it is the shortest time interval that has more than one data point for input data. For data points collected at every 6 months (i.e. from months 6 to 12, months 12 to 18, and months 18 to 24), each time interval consisted 2 model cycles. We assumed a constant transition probability in the 2 model cycles between each time interval: for instance, transition probabilities from months 6 to 9 and from months 9 to 12 are assumed identical, deriving from the data points from months 6 to 12. The proportion of patients transitioning from one health state to another one at each time point was grouped into a transition matrix. The transition matrices were then converted into hazards and transformed into transition probabilities by applying the appropriate time unit.

#### Costs

Cost data were not collected in CHIMES and CHIMES-E study. A literature search was conducted to obtain published cost data in a similar report format as our model. Lekander et al. reported post-stroke costs by stroke type (haemorrhagic and ischemic strokes), and stratified by functional disability in a format: mRS 0 to 2, mRS 3, mRS 4, mRS 5, and mRS 6 [15]. Ischemic stroke costs were extracted as this stroke type is more relevant to CHIMES and CHIMES-E study population.

Several assumptions were made for the cost computation to fit our model structure. First, the cost data was reported in mRS 0 to 2. So, in order to fit for health state mRS 0–1 in our model, we made an assumption that two thirds of the total costs in mRS 0–2 were incurred by mRS 2 and one third by mRS 0 to 1. A lower weight was applied to mRS 0–1 as this health state has lower functional disability compared to mRS 2, the latter of which may lead to higher post-stroke cost. Secondly, although the study reported costs for mRS 6 which is defined as the state of death, we assumed zero cost for this health state in the model. This is because costs are associated in other health states before patients die, those costs are already accrued in other health states in the Markov model. Therefore, no cost was associated with death in this study.

Lekander et al. reported costs after stroke from a societal perspective [15]. Our study undertook a health system perspective; therefore, only direct costs were extracted for our analyses. The costs were time dependent as the authors categorised the costs by first and second years. Hence, we introduced a time-dependent element into our models in accordance with the published data.

The study reported costs in Swedish Krona (SEK) in year 2016. We applied the consumer price index of Sweden to adjust the cost data to year 2022 [16]. Next, we converted Swedish Krona (SEK) to Euro (€) as a common currency (Euro 1 = USD 1.08).

#### Utilities

The EuroQOL five dimensions (EQ-5D) questionnaire is a commonly used health-related quality-of-life instrument for the outcome measure in health economic evaluations. Such data was not available from the CHIMES and CHIMES-E study. As a result, we searched for published literature on utility scores stratified by mRS. One study pooled EQ-5D-3 L utility scores from multiple neighbouring countries such as Malaysia, Philippines and Singapore, and applied Singapore’s value set to the pooled data [17]. The utility scores used are based on the VISTA (Virtual International Stroke Trials Archive) study, which collects data from completed clinical trials [17]. The pooled utility scores are relevant given that the pooled populations overlapped with some of the populations from CHIMES and CHIMES-E, which consisted of Hong Kong, Malaysia, Philippines, Singapore, Sri Lanka and Thailand. The published EQ-5D utilities were reported individually for each mRS score. We averaged the utilities of mRS 0 and mRS 1 to feed into the utility score for health state mRS 0–1. The utility score for death is zero.

#### Cost-utility analyses

We included patients having suffered a stroke of intermediate severity with baseline NIHSS score 8 to 14 (b-NIHSS 8–14) as the study population in the base-case analysis. To account for heterogeneity of patient characteristics, subgroup analyses were explored. Several subgroups with poor prognostic factors were included in the analyses [18–20]: patients with b-NIHSS 10–14, stroke onset to first dose greater than 48 h, and rehabilitation during the first 3 months. The differences in functional recovery between base-case population and subgroups with poor prognostic factors are reflected in transition probabilities.

Sensitivity analyses were conducted to check for the robustness of the results pertaining to parameter uncertainty. One-way deterministic sensitivity analysis was employed to check for the impact of the extreme values (low and high values) of input parameters, individually, to the results. In addition, probabilistic sensitivity analysis using Monte Carlo simulation was employed to randomise the input parameters from its appropriate distributions simultaneously for 10,000 runs to simulate the results with different combinations of input parameters drawn [21]. The results from Monte Carlo simulation are depicted in a cost-effectiveness acceptability curve (CEAC), where it computes the probability of each strategy of being cost-effective across a range of willingness-to-pay (WTP) thresholds. Both deterministic and probabilistic sensitivity analyses were performed for base-case and all subgroups.

We followed the Consolidated Health Economics Evaluation Reporting Standards (CHEERS) guidelines to guide us in the Reporting checklist for economic evaluation of health interventions based on the CHEERS guidelines (Supplemental Table 1) [22]. Statistical analyses and cost-utility analyses were conducted in RStudio Desktop Pro (version 2022.7.1.554.3) and TreeAge Pro (version 2022, R2 Healthcare), respectively [23, 24].

## Results

Baseline characteristics of base-case population presented in Table 1 are the same as those reported in the time-saving analysis publication [12].

**Table 1:** Baseline characteristics of base-case population (b-NIHSS 8–14)

Characteristics	Base-case analysis population (b-NIHSS 8–14)		
	MLC601	Placebo	*P*-value
N	287	261	0.44
Age (years), mean (SD)	61.4 (11.0)	63.1 (11.4)	0.08
• Age > 60 years	145 (50.5)	155 (59.4)	
Gender, male, n (%)	166 (57.8)	160 (61.3)	0.43
OTT: Time from stroke onset to study treatment (hours), mean (SD)	49.8 (17.3)	50.0 (17.4)	0.89
• Time from stroke onset to study treatment > 48 h, n (%)	148 (51.6)	145 (55.6)	
b-NIHSS total score (15 items), median (Q1, Q3)	10.0 (9.0, 12.0)	10.0 (9.0, 12.0)	0.68
mRS score at day 10, median (Q1, Q3)	4.0 (3.0, 4.0)	4.0 (3.0, 4.0)	0.90
Rehabilitation, n (%)	123 (42.9)	121 (46.4)	0.44
Vascular history and risk factors, n (%)			
• Ischaemic cerebrovascular disease	25 (8.7)	21 (8.0)	0.88
• Ischaemic cardiovascular disease	11 (3.8)	14 (5.4)	0.42
• Peripheral vascular disease	3 (1.0)	1 (0.4)	0.63
• Hypertension	238 (82.9)	212 (81.2)	0.66
• Diabetes	82 (28.6)	77 (29.5)	0.85
• Hyperlipidaemia	114 (39.7)	105 (40.2)	0.93
• Smoking	124 (43.2)	108 (41.4)	0.73
• Habitual alcohol intake	77 (26.8)	62 (23.8)	0.43

*b-NIHSS* baseline NIH stroke scale, *SD* Standard deviation, *mRS* modified Rankin Scale

*P*-values were obtained to compare two treatments groups (MLC601 vs. Placebo) using the two-sample t-test for continuous variables, Fisher’s exact test for categorical variables, and Wilcoxon rank-sum test for discrete variables

In subgroups with poor prognostic factors, both MLC601 and placebo groups are well-balanced with comparable baseline characteristics with poor prognosis factors such as NIHSS 10–14 and stroke onset to first dose greater than 48 h (Additional Tables 2A and 2B), except for the subgroup of rehabilitation during first 3 months (Additional Table 2C), where the proportion of males in placebo group (71.9%) is significantly higher than MLC601 group (56.9%).

### Cost-utility analyses

Cost data and utility scores used in the analyses are available in Supplemental Table 3. Transition probabilities were derived separately for base-case and subgroup analyses, and the data inputs are detailed in Supplemental Table 4.

The results of base-case group and subgroup analyses are shown in Table 2. Base-case sample results indicate that MLC601 is cost-saving, being €5,080 (USD 5,510.78) cheaper and more effective (0.45 incremental QALY gained) compared to the placebo group, resulting an ICER of -€11,352.50 (-USD 12,312.92) per QALY gained. Treatment intervention with MLC601 is therefore the dominant strategy. All subgroups show consistent results with different magnitude in the incremental cost saved and QALY gained (Table 2).

**Table 2:** Results from cost-utility analyses for base-case and subgroup populations

Strategy	Cost (€)	Incremental cost (€)	QALY	Incremental QALY	ICER (€/QALY)
*Base-case*					
Baseline NIHSS 8-14					
Placebo	38,346		4.12		
MLC601	33,266	-5,080	4.57	0.45	-11,352.50
*Subgroups*					
Baseline NIHSS 10-14					
Placebo	42,292		3.76		
MLC601	37,917	-4,375	4.26	0.50	-8,714.88
Stroke onset to first dose >48 hours					
Placebo	43,989		3.67		
MLC601	34,759	-9,230	4.35	0.67	-13,728.18
Rehabilitation during first 3 months					
Placebo	45,329		3.58		
MLC601	39,677	-5,652	4.28	0.69	-8,142.71

*NIHSS* NIH stroke scale, *QALY* quality-adjusted life-year, *ICER* incremental cost-effectiveness ratio

Base-case and all subgroups have negative ICER values, stemming from negative incremental cost comparing MLC601 to placebo. The negative incremental costs and positive incremental QALY gained of MLC601 indicate MLC601 is dominant and therefore cost-effective

**Fig. 3:**
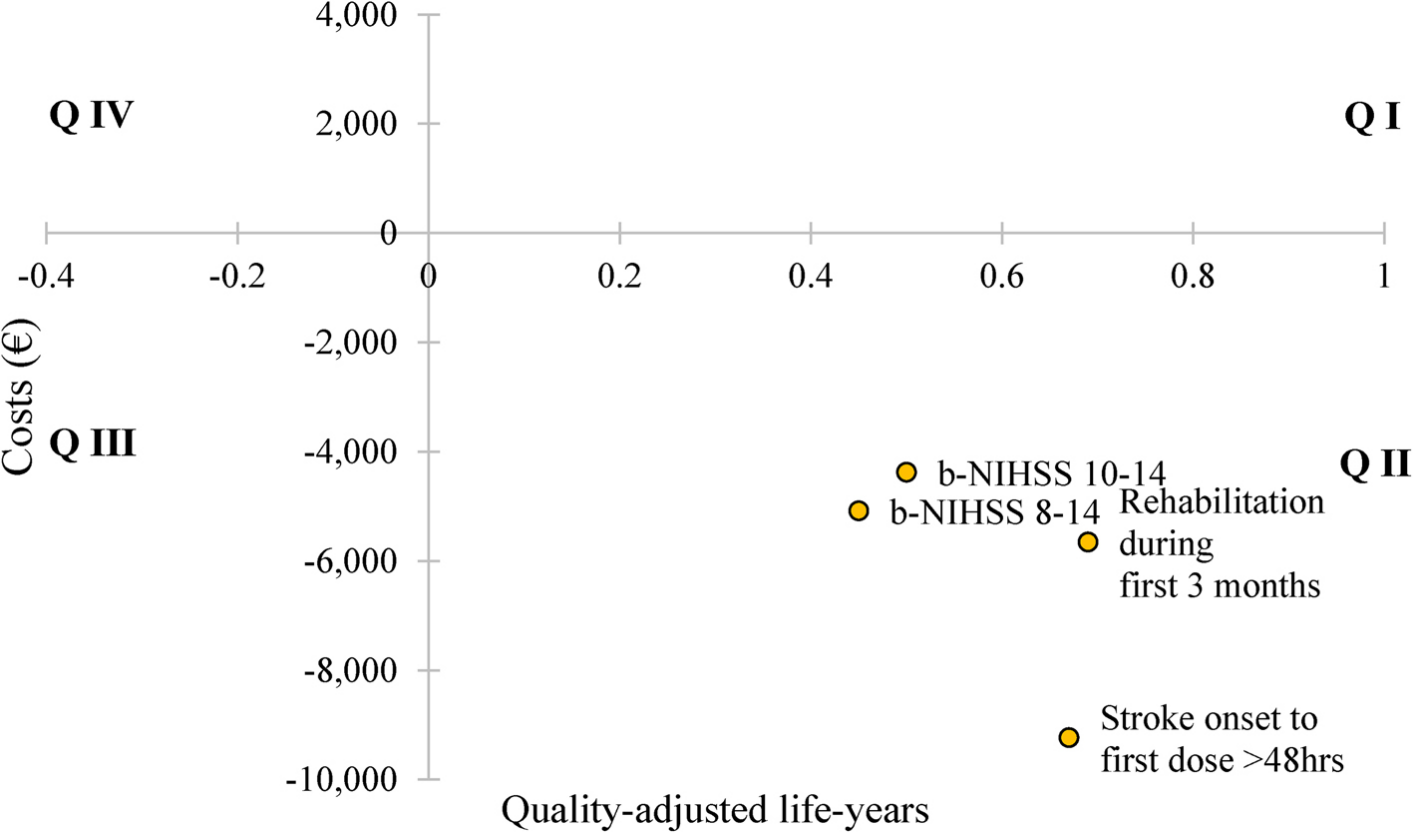
Cost-effectiveness plane for base-case group and subgroup analyses. b-NIHSS: baseline NIH stroke scale. X-axis: Incremental quality-adjusted life-years (QALYs) gained of MLC601 compared to placebo. Y-axis: Incremental cost of MLC601 compared to placebo in Euro. There are four quadrants in the cost-effectiveness plane, namely quadrant I (Q I), quadrant II (Q II), quadrant III (Q III), and quadrant IV (Q IV). If the incremental cost-effectiveness ratio (ICER), calculated by incremental cost divided by incremental QALYs gained, lies in Q II or Q IV, the choice between the strategies is obvious. In Q II, there is negative incremental cost in the MLC601 group, indicating a cost-saving, and positive incremental QALYs gained signifying MLC601 will dominate placebo. Inversely, if the ICER sits in Q IV, MLC601 costs more and has negative incremental QALYs gained, MLC601 will be dominated by placebo. For ICERs which lie in either Q I or Q III, a willingness-to-pay threshold is required to determine the cost-effectiveness. The ICERs for base-case and all subgroups are situated in Q II, indicating MLC601 is dominant and the preferred strategy

Figure 3 shows the cost-effectiveness plane of base-case and all subgroup analyses. All results are situated in quadrant II, indicating MLC601 is a dominant strategy with lower cost and higher QALY gained. Amongst all subgroups, the subgroup with stroke onset to first dose greater than 48 h has the highest incremental cost saved per QALY magnitude, with ICER -€13,352.50 (-USD 14,482.12) per QALY gained (€9,230; USD 10,012.70 saved and 0.67 QALY gained), followed by subgroup b-NIHSS 10 to 14 with ICER -€8,714.88 (-USD 9,453.03) per QALY gained (€4,375; USD 4,744.69 saved and 0.50 QALY gained) and lastly, subgroup rehabilitation during first 3 months with ICER -€8,142.71 (-USD 8,831.58) per QALY gained (€5,652; USD 6,128.46 saved and 0.69 QALY gained).

### Sensitivity analyses

**Fig. 4:**
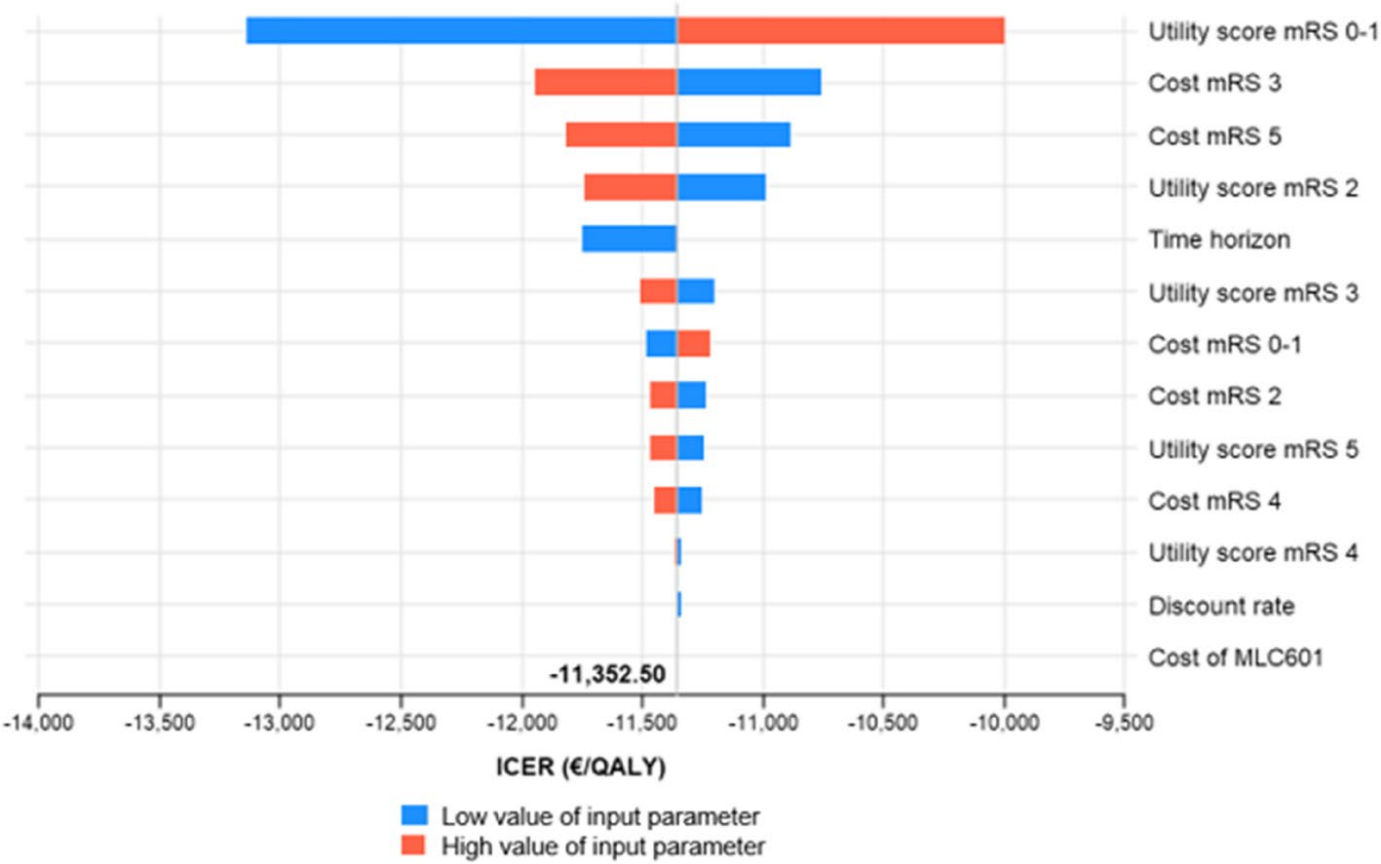
Tornado diagram for base-case population (b-NIHSS 8–14) analysis. A Tornado diagram is used to present the results of one-way sensitivity analysis. A list of input parameters was varied one by one, by the lower and higher bound of the mean value of the parameters. The difference of ICERs estimated by varying the extreme values of input parameter was calculated, and arranged from the largest to the lowest difference. The largest difference is shown at the top of the Tornado diagram, and followed by the parameters with smaller difference in ICERs. The Tornado diagram, as suggested by its name, always has a shape of inverted triangle symbolizing the shape of a tornado. The extreme values (low and high) of each input parameter resulted in negative ICERs signifying the uncertainty around the mean values did not have huge impact to the result

In Fig. 4, a Tornado diagram illustrates the result from one-way deterministic sensitivity analysis. Extreme values of input parameters did not have a large impact to the model results, and MLC601 remains as a cost-effective strategy compared to placebo. Tornado diagrams for subgroups with poor prognosis factors have the same conclusion and are available in Supplemental Fig. 1.

**Fig. 5:**
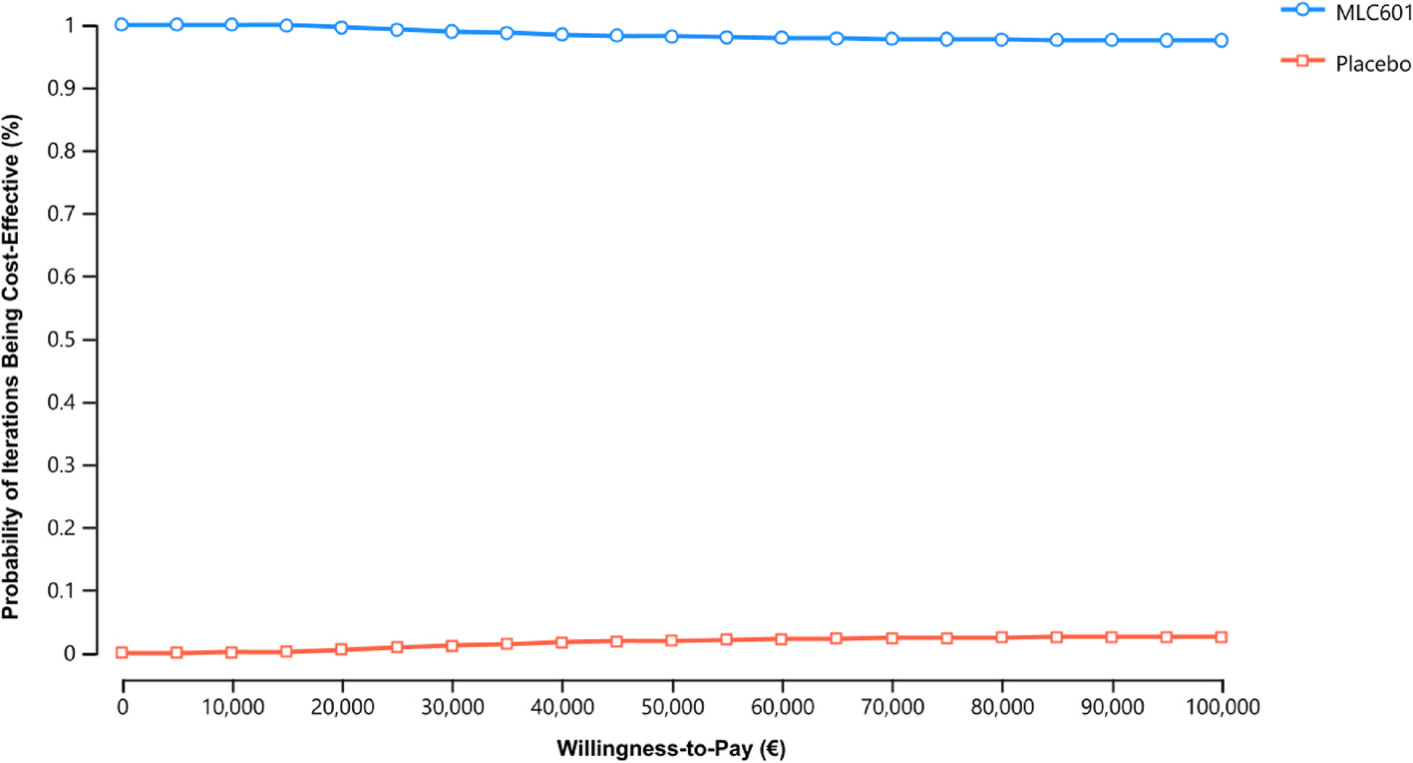
Cost-effectiveness acceptability curve for base-case population (b-NIHSS 8–14) analysis. The x-axis shows a range of willingness-to-pay (WTP) threshold in Euro and the y-axis represents the probability of the strategy is being cost-effective from Monte-Carlo simulation. Cost-effectiveness acceptability curve shows the cost-effectiveness results from Monte-Carlo simulation (10,000 iterations) against a range of WTP threshold. The blue line represents MLC601, which has always been the preferred strategy, with close to 100% of iterations being cost-effective, across the range of WTP threshold. At a range of WTP threshold from zero to €100,000, MLC601 appears to be cost-effective compared to placebo

The base-case results from Monte Carlo simulation are graphed in a CEAC (Fig. 5). At least 95% of the 10,000 simulations show that MLC601 is being cost-effective over a range of WTP threshold in all models. CEACs for subgroups with poor prognosis factors are provided in Supplemental Fig. 2.

Results from sensitivity analyses are consistent with the results from the models, indicating that the results are robust.

## Discussion

The cost-utility analysis shows that MLC601 is cost-effective for post-stroke functional recovery in patients with AIS of intermediate severity (b-NIHSS 8–14). MLC601 incurs less costs and improves patient’s quality-of-life compared to placebo, with €11,352.50 (USD 12,312.92) saved per QALY from a health system perspective. When exploring subgroups with poor prognosis factors, the results show that MLC601 is dominant in all subgroups.

This is the first economic evaluation to assess the cost-effectiveness of MLC601 as an add-on to the standard of care in post-stroke recovery. In the present analysis, we compared the cost-effectiveness of MLC601 versus placebo, using the data from a well-conducted multicentre acute stroke clinical study programmed with a long follow-up period [10, 11]. Many papers have been published regarding the efficacy of MLC601 versus placebo, but little is known about the cost-effectiveness of implementation of the treatment to the health system.

The study treatment (MLC601) was administered for only 3 months, but the benefit in functional recovery extended to two years [11]. The treatment costs of 3 months were relatively low compared to the costs associated with more severe functional disability (i.e., mRS score 3–5). By improving a sustained functional recovery to two years with only 3 months of treatment, the treatment group appears to have achieved cost saving with faster functional recovery and better quality of life assessed by EQ-5D-3 L utility scores stratified by mRS scores.

The World Health Organization recommends a threshold 1 to 3 times of country’s gross domestic product (GDP) per capita as a guide in determining cost-effectiveness of health interventions [25]. However, we did not choose a specific WTP threshold as our study consisted of multinational data (i.e., Hong Kong, Malaysia, Philippines, Singapore, Sri Lanka and Thailand), and therefore there was no unique threshold from all participating countries. Instead, CEACs produced in the sensitivity analyses were used to guide an assessment of the cost-effectiveness of MLC601 over a range of WTP thresholds. It is also important to note that the absence of WTP threshold does not affect drawing conclusion from the results as MLC601 is dominant in all analyses.

There were several limitations to the study. Firstly, as explained in the Method section, there was no economic evaluation planned alongside the CHIMES and CHIMES-E study. Therefore, we did not have cost and utility score data collected from the enrolled patients. Published data were sourced for data input in our model. We extracted the costs from a study conducted in Sweden, as the distribution of post-stroke care costs comes with a reporting format similar to our model [15]. Based on our literature review, this was the only study reporting costs stratified by mRS scores at 1 and 2 years with a time-dependent value in patients after an ischemic stroke. However, the potential bias from this data source is that it was based on the costs in Sweden, a high-income country, while the patients in the CHIMES and CHIMES-E study were recruited from various countries in South East Asia including lower/upper middle-income countries such as Philippines, Sri Lanka, and Thailand. Using cost data from a high-income country in the model might not be a good representation of the real-world cost in all the participating countries. In a systematic review of economic burden of stroke, a major issue synthesizing published cost-of-illness studies was methodological heterogeneity in costing, making comparisons difficult or even impossible [26]. In addition, the literature on the economic burden of stroke mainly concerns high-income countries, with the data available in low-/upper-middle-income countries being rather sparse.

The lack of prospectively collected data also applies to the health utility data. We extracted the utility scores from the VISTA study, which consisted of patient-level data from completed stroke clinical trials [17]. The authors reported post-stroke health utility scores by merging several neighbouring countries and applying a single value to all these countries. Thus, the Hong Kong data reported in this study were pooled with those of China and Taiwan, and subsequently analysed using China’s value set. The combined utility scores of these are generally higher than those of Malaysia, Philippines, and Singapore, making the utility scores from these three countries (Malaysia, Philippines, and Singapore) used in our analysis conservative estimates. Although Hong Kong was one of the study sites, its utility scores were not included in our analysis, but since the utility scores reported in Hong Kong are higher than those of the three countries used in our analysis, the use of combined data from China, Hong Kong and Taiwan would not change the conclusion of the results. Having said that, the baseline characteristics of the population in the VISTA study appear to be older with mean age of 68.8 years (SD = 12.6) while our study population is younger with mean age of 61.4 years (SD = 11.0) and 63.1 years (SD = 11.4), in MLC601 and placebo groups, respectively. Besides, there were some selection criteria applied to our study population, as described in Methods section, that might not be comparable to the VISTA study population. This could lead to selection bias as the data from the VISTA study might not be representative of our study population.

The population included in this analysis was a subset from the original CHIMES study, with increased stroke severity at baseline measured b-NIHSS 8–14. Although about only half (55%) of the total sample (607 among 1109 patients) were included in this analysis, the baseline characteristics of both MLC601 and placebo groups were still comparable (Table 1). Including only increased stroke severity subjects in the study may be subject for selection bias as it would potentially inflate the overall benefit of MLC601. Past studies have demonstrated that patients with poorer prognosis for post-stroke recovery were more likely to benefit from MLC601 treatment [18–20]. Therefore, caution must be taken when interpreting the results from this analysis.

Another limitation of the study is the conceptualization of the model structure and the assumptions made. We modelled the stroke recovery based solely on the clinical endpoint measured in mRS scores. Stroke has a complex recovery journey, hence using the changes in mRS scores to represent poststroke recovery may be an oversimplification. Discussion on the use of mRS and the different clinical endpoints for stroke trials have concluded that each measurement has its own advantages and disadvantages, hence more refined endpoints are needed for future stroke studies [27, 28]. Nevertheless, mRS is one of the commonly used clinical endpoints in clinical trials for stroke and in economic evaluations [13, 14, 29–31].

Furthermore, our model has a time horizon of 2 years, but stroke has long term impact [1]. Generally, the time horizon in economic evaluations should be sufficiently long to capture the significant differences in costs and effectiveness between the intervention and comparator groups [22]. We did not choose a longer time horizon because there was limited data on the follow-up period beyond 2 years. Even though data extrapolation and assumptions could be applied to study the incremental differences between the two groups, we undertook a conservative approach by not modelling the long-term effect. Future research could focus on following up the population for long-term to shed some light in this area.

One of the assumptions made in this study was that death did not incur costs in our model, as opposed to what was reported in the cited Swedish paper [15]. The incidence of death was low and did not differ significantly between MLC601 and placebo groups. Therefore, the inclusion of death cost in the model will not change the conclusion of the results.

The patient pathways or management for stroke are complex and contextual to health systems. The analysis is post hoc and thus hypothesis generating, and confirmatory analyses should be conducted. Relevant local data and evidence should be adopted, whenever possible, and model structure could be modified to be representative of the stroke management in their own health system. We also suggest conducting further analyses to segregate countries by income groups supplementing with country-specific healthcare costs and utility data to better reflect the impact of treatment effect on long-term savings for stroke patients.

## Conclusions

This is the first cost-utility analysis undertaken using data from CHIMES and CHIMES-E to evaluate the cost-effectiveness of MLC601 in post-stroke recovery. The results of this study support that MLC601 on top of standard stroke care is a cost-effective intervention in post-stroke recovery of patients compared to placebo. However, this conclusion may not be generalized to other settings due to lack of local economic data. Further studies with country-specific data are needed to apply the methods of this study in their own decision-making context and clinical setting.

## Supplementary Information

Supplementary Material 1.

## Data Availability

All data generated or analysed during this study are included in this article, along with references to data from cited published studies. The database is not publicly available. The datasets analysed during the current study are available from C.L.H.C., the corresponding author, on reasonable request.

## Abbreviations

AIS: Acute ischemic stroke
CHEERS: Consolidated Health Economics Evaluation Reporting Standards
CHIMES: CHInese Medicine Neuroaid Efficacy on Stroke recovery
CHIMES-E: CHInese Medicine Neuroaid Efficacy on Stroke recovery - Extension
€: Euro
EQ-5D: EuroQOL five dimensions
GDP: Gross domestic product
ICER: Incremental cost-effectiveness ratio
MLC: Moleac
mRS: modified Rankin Score
b-NIHSS: Baseline NIH Stroke Scale
QALY(s): Quality-adjusted life-year(s)
SEK: Swedish Krona
USD: US Dollar
VISTA: Virtual International Stroke Trials Archive
WTP: Willingness-to-pay
