# Supplementary material for: Cost-effectiveness of MLC601 in post-stroke functional recovery compared with placebo - the CHIMES & CHIMES-E studies
Source: BMC Health Serv Res. 2024 Sep 27;24:1127. doi: 10.1186/s12913-024-11618-4 (PMC11430337; doi:10.1186/s12913-024-11618-4)
Supplement: Supplementary file 1 — Supplementary Material 1. [file 12913_2024_11618_MOESM1_ESM.docx]

# Supplementary Material

# Supplemental Table 1 Reporting checklist for economic evaluation of health interventions based on the CHEERS guidelines.

Supplemental Table 2 Baseline characteristics of subgroup baseline NIHSS 10 to 14.

Supplemental Table 3 Baseline characteristics of subgroup stroke onset to first dose more than 48 hours.

Supplemental Table 4 Baseline characteristics of subgroup rehabilitation during first 3 months.

Supplemental Table 5 Input parameters of cost-utility analysis.

Supplemental Table 6 Transition probabilities of base-case analysis (b-NIHSS 8-14).

Supplemental Table 7 Transition probabilities of subgroup baseline NIHSS 10 to 14.

Supplemental Table 8 Transition probabilities of subgroup stroke onset to first dose greater than 48 hours.

Supplemental Table 9 Transition probabilities of subgroup rehabilitation during first 3 months.

Supplemental Figure 1 Tornado diagram for subgroup baseline NIHSS 10 to 14.

Supplemental Figure 2 Tornado diagram for subgroup stroke onset to first dose greater than 48 hours.

Supplemental Figure 3 Tornado diagram for subgroup rehabilitation during first 3 months.

Supplemental Figure 4 Cost-effectiveness acceptability curve for subgroup baseline NIHSS 10 to 14.

Supplemental Figure 5 Cost-effectiveness acceptability curve for subgroup stroke onset to first dose greater than 48 hours.

Supplemental Figure 6 Cost-effectiveness acceptability curve for subgroup rehabilitation during first 3 months.

# Supplemental Table 1 Reporting checklist for economic evaluation of health interventions based on the CHEERS guidelines.

|  |  | Reporting Items | Page Number |
| --- | --- | --- | --- |
| **Title** | [#1](https://www.goodreports.org/reporting-checklists/cheers/info/#1) | Identify the study as an economic evaluation or use more specific terms such as “cost-effectiveness analysis”, and describe the interventions compared. | 1 |
| **Abstract** | [#2](https://www.goodreports.org/reporting-checklists/cheers/info/#2) | Provide a structured summary of objectives, perspective, setting, methods (including study design and inputs), results (including base case and uncertainty analyses), and conclusions | 3-4 |
| **Introduction** |  |  |  |
| Background and objectives | [#3](https://www.goodreports.org/reporting-checklists/cheers/info/#3) | Provide an explicit statement of the broader context for the study. Present the study question and its relevance for health policy or practice decisions | 5 |
| **Methods** |  |  |  |
| Target population and subgroups | [#4](https://www.goodreports.org/reporting-checklists/cheers/info/#4) | Describe characteristics of the base case population and subgroups analysed, including why they were chosen. | 6, 9-10 |
| Setting and location | [#5](https://www.goodreports.org/reporting-checklists/cheers/info/#5) | State relevant aspects of the system(s) in which the decision(s) need(s) to be made. | 5-6 |
| Study perspective | [#6](https://www.goodreports.org/reporting-checklists/cheers/info/#6) | Describe the perspective of the study and relate this to the costs being evaluated. | 7-9 |
| Comparators | [#7](https://www.goodreports.org/reporting-checklists/cheers/info/#7) | Describe the interventions or strategies being compared and state why they were chosen. | 7 |
| Time horizon | [#8](https://www.goodreports.org/reporting-checklists/cheers/info/#8) | State the time horizon(s) over which costs and consequences are being evaluated and say why appropriate. | 7 |
| Discount rate | [#9](https://www.goodreports.org/reporting-checklists/cheers/info/#9) | Report the choice of discount rate(s) used for costs and outcomes and say why appropriate | 7 |
| Choice of health outcomes | [#10](https://www.goodreports.org/reporting-checklists/cheers/info/#10) | Describe what outcomes were used as the measure(s) of benefit in the evaluation and their relevance for the type of analysis performed | 7-8 |
| Measurement of effectiveness | [#11a](https://www.goodreports.org/reporting-checklists/cheers/info/#11a) | Single study-based estimates: Describe fully the design features of the single effectiveness study and why the single study was a sufficient source of clinical effectiveness data | 5-6 |
| Measurement of effectiveness | [#11b](https://www.goodreports.org/reporting-checklists/cheers/info/#11b) | Synthesis-based estimates: Describe fully the methods used for identification of included studies and synthesis of clinical effectiveness data |  |
| Measurement and valuation of preference based outcomes | [#12](https://www.goodreports.org/reporting-checklists/cheers/info/#12) | If applicable, describe the population and methods used to elicit preferences for outcomes. | 9 |
| **Estimating resources and costs ** | [#13a](https://www.goodreports.org/reporting-checklists/cheers/info/#13a) | Single study-based economic evaluation: Describe approaches used to estimate resource use associated with the alternative interventions. Describe primary or secondary research methods for valuing each resource item in terms of its unit cost. Describe any adjustments made to approximate to opportunity costs |  |
| Estimating resources and costs | [#13b](https://www.goodreports.org/reporting-checklists/cheers/info/#13b) | Model-based economic evaluation: Describe approaches and data sources used to estimate resource use associated with model health states. Describe primary or secondary research methods for valuing each resource item in terms of its unit cost. Describe any adjustments made to approximate to opportunity costs. | 6-9 |
| Currency, price date, and conversion | [#14](https://www.goodreports.org/reporting-checklists/cheers/info/#14) | Report the dates of the estimated resource quantities and unit costs. Describe methods for adjusting estimated unit costs to the year of reported costs if necessary. Describe methods for converting costs into a common currency base and the exchange rate. | 8-9 |
| Choice of model | [#15](https://www.goodreports.org/reporting-checklists/cheers/info/#15) | Describe and give reasons for the specific type of decision analytical model used. Providing a figure to show model structure is strongly recommended. | 6-7, 21-22 (Figure 1 & 2) |
| Assumptions | [#16](https://www.goodreports.org/reporting-checklists/cheers/info/#16) | Describe all structural or other assumptions underpinning the decision-analytical model. | 8-10 |
| Analytical methods | [#17](https://www.goodreports.org/reporting-checklists/cheers/info/#17) | Describe all analytical methods supporting the evaluation. This could include methods for dealing with skewed, missing, or censored data; extrapolation methods; methods for pooling data; approaches to validate or make adjustments (such as half cycle corrections) to a model; and methods for handling population heterogeneity and uncertainty. | 7, 9-10 |
| **Results** |  |  |  |
| Study parameters | [#18](https://www.goodreports.org/reporting-checklists/cheers/info/#18) | Report the values, ranges, references, and, if used, probability distributions for all parameters. Report reasons or sources for distributions used to represent uncertainty where appropriate. Providing a table to show the input values is strongly recommended. | 25 (Table 2) |
| Incremental costs and outcomes | [#19](https://www.goodreports.org/reporting-checklists/cheers/info/#19) | For each intervention, report mean values for the main categories of estimated costs and outcomes of interest, as well as mean differences between the comparator groups. If applicable, report incremental cost-effectiveness ratios. | 11, 26 (Table 3) |
| Characterizing uncertainty | [#20a](https://www.goodreports.org/reporting-checklists/cheers/info/#20a) | Single study-based economic evaluation: Describe the effects of sampling uncertainty for the estimated incremental cost and incremental effectiveness parameters, together with the impact of methodological assumptions (such as discount rate, study perspective). | n/a  model-based study based on single RCT |
| Characterizing uncertainty | [#20b](https://www.goodreports.org/reporting-checklists/cheers/info/#20b) | Model-based economic evaluation: Describe the effects on the results of uncertainty for all input parameters, and uncertainty related to the structure of the model and assumptions. | 12, 28-29 (Figure 4 & 5) |
| Characterizing heterogeneity | [#21](https://www.goodreports.org/reporting-checklists/cheers/info/#21) | If applicable, report differences in costs, outcomes, or cost effectiveness that can be explained by variations between subgroups of patients with different baseline characteristics or other observed variability in effects that are not reducible by more information. | 10-12, Supplemental Table 1-3, Supplemental Figure 1-3 |
| **Discussion** |  |  |  |
| Study findings, limitations, generalizability, and current knowledge | [#22](https://www.goodreports.org/reporting-checklists/cheers/info/#22) | Summarise key study findings and describe how they support the conclusions reached. Discuss limitations and the generalisability of the findings and how the findings fit with current knowledge. | 12- 14 |
| **Other** |  |  |  |
| Source of funding | [#23](https://www.goodreports.org/reporting-checklists/cheers/info/#23) | Describe how the study was funded and the role of the funder in the identification, design, conduct, and reporting of the analysis. Describe other non-monetary sources of support | 15 |
| Conflict of interest | [#24](https://www.goodreports.org/reporting-checklists/cheers/info/#24) | Describe any potential for conflict of interest of study contributors in accordance with journal policy. In the absence of a journal policy, we recommend authors comply with International Committee of Medical Journal Editors recommendations | 16 |

Supplemental Table 2 Baseline characteristics of subgroup baseline NIHSS 10 to 14.

| Characteristics | MLC601 | Placebo | p-value |
| --- | --- | --- | --- |
| N | 175 | 153 |  |
| Age (years), mean (SD) | 61.1 (11.0) | 62.6 (11.5) | 0.239 |
| Gender, male, n (%) | 111 (63.4) | 95 (62.1) | 0.892 |
| Ethnicity, n (%) |  |  | 0.558 |
| Filipino | 108 (61.7) | 95 (62.1) |  |
| Chinese | 30 (17.1) | 35 (22.9) |  |
| Thai | 16 (9.1) | 8 (5.2) |  |
| Malay | 12 (6.9) | 7 (4.6) |  |
| Indian | 3 (1.7) | 2 (1.3) |  |
| Others | 6 (3.4) | 6 (3.9) |  |
| Stroke onset to first dose (hours), mean (SD) | 49.5 (17.0) | 52.3 (16.9) | 0.145 |
| Stroke onset to first dose >48hours, n (%) | 91 (52.6) | 96 (62.7) | 0.083 |
| Rehabilitation, n (%) | 76 (43.4) | 72 (47.1) | 0.584 |

NIHSS: NIH stroke scale; SD: standard deviation

P-values were obtained to compare two treatments groups (MLC601 vs. Placebo) using the two-sample t-test for continuous variables, and Chi-squared test for categorical variables.

Supplemental Table 3 Baseline characteristics of subgroup stroke onset to first dose more than 48 hours.

| Characteristics | MLC601 | Placebo | p-value |
| --- | --- | --- | --- |
| N | 148 | 145 |  |
| Age (years), mean (SD) | 62.8 (10.5) | 62.7 (11.3) | 0.965 |
| Gender, male, n (%) | 81 (54.7) | 88 (60.7) | 0.361 |
| Ethnicity, n (%) |  |  | 0.560 |
| Filipino | 95 (64.2) | 85 (58.6) |  |
| Chinese | 25 (16.9) | 36 (24.8) |  |
| Thai | 17 (11.5) | 12 (8.3) |  |
| Malay | 7 (4.7) | 6 (4.1) |  |
| Indian | 1 (0.7) | 2 (1.4) |  |
| Others | 3 (2.0) | 4 (2.8) |  |
| Stroke onset to first dose (hours), mean (SD) | 64.0 (8.0) | 63.4 (8.4) | 0.498 |
| Stroke onset to first dose >48hours, n (%) | 160 (100.0) | 155 (100.0) | N/A |
| Rehabilitation, n (%) | 61 (41.2) | 73 (50.3) | 0.147 |

SD: standard deviation

P-values were obtained to compare two treatments groups (MLC601 vs. Placebo) using the two-sample t-test for continuous variables, and Chi-squared test for categorical variables.

Supplemental Table 4 Baseline characteristics of subgroup rehabilitation during first 3 months.

| Characteristics | MLC601 | Placebo | p-value |
| --- | --- | --- | --- |
| N | 123 | 121 |  |
| Age (years), mean (SD) | 62.2 (10.8) | 63.5 (11.2) | 0.359 |
| Gender, male, n (%) | 70 (56.9) | 87 (71.9) | 0.021 |
| Ethnicity, n (%) |  |  | 0.661 |
| Filipino | 48 (39.0) | 44 (36.4) |  |
| Chinese | 47 (38.2) | 50 (41.3) |  |
| Thai | 4 (3.3) | 8 (6.6) |  |
| Malay | 11 (8.9) | 11 (9.1) |  |
| Indian | 3 (2.4) | 3 (2.5) |  |
| Others | 10 (8.1) | 5 (4.1) |  |
| Stroke onset to first dose (hours), mean (SD) | 49.5 (16.9) | 51.4 (16.6) | 0.378 |
| Stroke onset to first dose >48hours, n (%) | 61 (50.4) | 73 (60.3) | 0.155 |
| Rehabilitation, n (%) | 123 (100.0) | 121 (100.0) | N/A |

SD: standard deviation

P-values were obtained to compare two treatments groups (MLC601 vs. Placebo) using the two-sample t-test for continuous variables, and Chi-squared test for categorical variables.

Supplemental Table 5 Input parameters of cost-utility analysis.

| Variables | Mean (SD) | Low | High | Distribution | Source |
| --- | --- | --- | --- | --- | --- |
| Intervention cost (3 months) | €1,671.00 | - | - | - | Manufacturer |
| Costs |  |  |  |  | 13 |
| mRS 0-1 | €4,765.33 (243.13) ^#^  €1,691.00 (86.28) ^&^ | €4,288.80 ^#^  €1,521.90 ^&^ | €5,241.86 ^#^  €1,860.10 ^&^ | Gamma |  |
| mRS 2 | €9,530.66 (486.26) ^#^  €3,382.00 (172.55) ^&^ | €8,577.59 ^#^  €3,043.80 ^&^ | €10,483.72 ^#^  €3,720.20 ^&^ | Gamma |  |
| mRS 3 | €41,458.87 (2,115.25) ^#^  €41,110.68 (2,097.48) ^&^ | €37,312.98 ^#^  €36,999.61 ^&^ | €45,604.75 ^#^  €45,221.75 ^&^ | Gamma |  |
| mRS 4 | €71,372.96 (3,641.48) ^#^  €85,909.94 (4,383.16) ^&^ | €64,235.67 ^#^  €77,318.95 ^&^ | €78,510.26 ^#^  €94,500.94 ^&^ | Gamma |  |
| mRS 5 | €93,468.20 (4,768.79) ^#^  €90,692.02 (4,627.14) ^&^ | €84,121.38 ^#^  €81,622.82 ^&^ | €102,815.02 ^#^  €99,761.22 ^&^ | Gamma |  |
|  |  |  |  |  |  |
| Utilities |  |  |  |  | 15 |
| mRS 0-1 | 0.89 (0.21) | 0.80 | 0.97 | Beta |  |
| mRS 2 | 0.47 (0.26) | 0.42 | 0.52 | Beta |  |
| mRS 3 | 0.24 (0.33) | 0.22 | 0.26 | Beta |  |
| mRS 4 | -0.23 (0.29) | -0.25 | -0.21 | Normal |  |
| mRS 5 | -0.48 (0.17) | -0.53 | -0.43 | Normal |  |

SD: standard deviation; mRS: modified Rankin Scale

^#^: First year cost data

^&^: Second year cost data

Cost data are reported annually but converted to fit the cycle length of 3 months in the models.

Utility scores are reported as 3-month post-stroke health utilities, no adjustment was applied to fit the model’s cycle length.

Supplemental Table 6 Transition probabilities of base-case analysis (b-NIHSS 8-14).

| Transition from | MLC601 | Placebo | Source |
| --- | --- | --- | --- |
| mRS 0-1 to 2 | 0 ^α^  0.0694 ^β^  0.0111 ^γ, δ^  0.0053 ^ε, ζ^  0.0053 ^η, θ^ | 0 ^α^  0.0417 ^β^  0.0184 ^γ, δ^  0.0086 ^ε, ζ^  0.0078 ^η, θ^ | 11,12 |
| mRS 0-1 to 3 | 0 ^α^  0.0278 ^β^  0.0111 ^γ, δ^  0.0106 ^ε, ζ^  0.0053 ^η, θ^ | 0 ^α^  0.0417 ^β^  0 ^γ, δ^  0.0086 ^ε, ζ^  0 ^η, θ^ |  |
| mRS 0-1 to 4 | 0 ^α^  0 ^β^  0 ^γ, δ^  0 ^ε, ζ^  0.0107 ^η, θ^ | 0 ^α^  0.0208 ^β^  0 ^γ, δ^  0 ^ε, ζ^  0.0078 ^η, θ^ |  |
| mRS 0-1 to 5 | 0 ^α^  0 ^β^  0.0055 ^γ, δ^  0 ^ε, ζ^  0 ^η, θ^ | 0 ^α^  0.0208 ^β^  0 ^γ, δ^  0 ^ε, ζ^  0 ^η, θ^ |  |
| mRS 0-1 to 6 | 0 ^α^  0 ^β^  0 ^γ, δ^  0.0053 ^ε, ζ^  0 ^η, θ^ | 0 ^α^  0 ^β^  0 ^γ, δ^  0 ^ε, ζ^  0 ^η, θ^ |  |
| mRS 2 to 0-1 | 0.5926 ^α^  0.3115 ^β^  0.0756 ^γ, δ^  0.0385 ^ε, ζ^  0.0455 ^η, θ^ | 0.5429 ^α^  0.1852 ^β^  0.0433 ^γ, δ^  0.0856 ^ε, ζ^  0.0287 ^η, θ^ |  |
| mRS 2 to 3 | 0.0370 ^α^  0.1148 ^β^  0.0091 ^γ, δ^  0.0583 ^ε, ζ^  0.0225 ^η, θ^ | 0.0571 ^α^  0.1481 ^β^  0.0433 ^γ, δ^  0.0249 ^ε, ζ^  0.0095 ^η, θ^ |  |
| mRS 2 to 4 | 0 ^α^  0 ^β^  0.0184 ^γ, δ^  0.0095 ^ε, ζ^  0.0112 ^η, θ^ | 0.0286 ^α^  0.0370 ^β^  0 ^γ, δ^  0.0165 ^ε, ζ^  0.0095 ^η, θ^ |  |
| mRS 2 to 5 | 0 ^α^  0 ^β^  0 ^γ, δ^  0 ^ε, ζ^  0 ^η, θ^ | 0 ^α^  0 ^β^  0 ^γ, δ^  0.0082 ^ε, ζ^  0.0095 ^η, θ^ |  |
| mRS 2 to 6 | 0 ^α^  0.0164 ^β^  0.0091 ^γ, δ^  0 ^ε, ζ^  0 ^η, θ^ | 0 ^α^  0 ^β^  0.0085 ^γ, δ^  0.0082 ^ε, ζ^  0 ^η, θ^ |  |
| mRS 3 to 0-1 | 0.4937 ^α^  0.0638 ^β^  0.0206 ^γ, δ^  0.0120 ^ε, ζ^  0.0112 ^η, θ^ | 0.4262 ^α^  0.0161 ^β^  0 ^γ, δ^  0 ^ε, ζ^  0.0095 ^η, θ^ |  |
| mRS 3 to 2 | 0.3038 ^α^  0.1915 ^β^  0.0853 ^γ, δ^  0.0241 ^ε, ζ^  0.0455 ^η, θ^ | 0.3279 ^α^  0.2742 ^β^  0.0900 ^γ, δ^  0.0703 ^ε, ζ^  0.0385 ^η, θ^ |  |
| mRS 3 to 4 | 0.0253 ^α^  0.1064 ^β^  0 ^γ, δ^  0.0488 ^ε, ζ^  0.0112 ^η, θ^ | 0.0328 ^α^  0.0645 ^β^  0.0237 ^γ, δ^  0.0085 ^ε, ζ^  0.0095 ^η, θ^ |  |
| mRS 3 to 5 | 0.0127 ^α^  0 ^β^  0 ^γ, δ^  0 ^ε, ζ^  0 ^η, θ^ | 0.0164 ^α^  0 ^β^  0 ^γ, δ^  0 ^ε, ζ^  0.0095 ^η, θ^ |  |
| mRS 3 to 6 | 0 ^α^  0 ^β^  0 ^γ, δ^  0 ^ε, ζ^  0 ^η, θ^ | 0 ^α^  0 ^β^  0.0078 ^γ, δ^  0.0085 ^ε, ζ^  0.0095 ^η, θ^ |  |
| mRS 4 to 0-1 | 0.2000 ^α^  0.0303 ^β^  0 ^γ, δ^  0 ^ε, ζ^  0 ^η, θ^ | 0.1069 ^α^  0.0370 ^β^  0 ^γ, δ^  0 ^ε, ζ^  0 ^η, θ^ |  |
| mRS 4 to 2 | 0.2483 ^α^  0.0606 ^β^  0 ^γ, δ^  0.0202 ^ε, ζ^  0 ^η, θ^ | 0.2672 ^α^  0.1481 ^β^  0 ^γ, δ^  0 ^ε, ζ^  0.0282 ^η, θ^ |  |
| mRS 4 to 3 | 0.3172 ^α^  0.2121 ^β^  0.0202 ^γ, δ^  0.0202 ^ε, ζ^  0.0194 ^η, θ^ | 0.4198 ^α^  0.3333 ^β^  0.1271 ^γ, δ^  0.0318 ^ε, ζ^  0.0282 ^η, θ^ |  |
| mRS 4 to 5 | 0.0069 ^α^  0.0909 ^β^  0.0202 ^γ, δ^  0.0408 ^ε, ζ^  0 ^η, θ^ | 0 ^α^  0.0370 ^β^  0.0742 ^γ, δ^  0 ^ε, ζ^  0 ^η, θ^ |  |
| mRS 4 to 6 | 0 ^α^  0 ^β^  0 ^γ, δ^  0 ^ε, ζ^  0.0194 ^η, θ^ | 0 ^α^  0 ^β^  0 ^γ, δ^  0.0318 ^ε, ζ^  0.0282 ^η, θ^ |  |
| mRS 5 to 0-1 | 0 ^α^  0 ^β^  0 ^γ, δ^  0 ^ε, ζ^  0 ^η, θ^ | 0 ^α^  0 ^β^  0 ^γ, δ^  0 ^ε, ζ^  0 ^η, θ^ |  |
| mRS 5 to 2 | 0 ^α^  0 ^β^  0 ^γ, δ^  0 ^ε, ζ^  0 ^η, θ^ | 0 ^α^  0 ^β^  0 ^γ, δ^  0 ^ε, ζ^  0 ^η, θ^ |  |
| mRS 5 to 3 | 0.1429 ^α^  0 ^β^  0 ^γ, δ^  0.1056 ^ε, ζ^  0 ^η, θ^ | 0.2000 ^α^  0.2500 ^β^  0 ^γ, δ^  0 ^ε, ζ^  0 ^η, θ^ |  |
| mRS 5 to 4 | 0.8571 ^α^  0 ^β^  0 ^γ, δ^  0 ^ε, ζ^  0.1056 ^η, θ^ | 0.4000 ^α^  0 ^β^  0 ^γ, δ^  0.0871 ^ε, ζ^  0 ^η, θ^ |  |
| mRS 5 to 6 | 0 ^α^  0 ^β^  0.2254 ^γ, δ^  0.1056 ^ε, ζ^  0 ^η, θ^ | 0 ^α^  0.2500 ^β^  0 ^γ, δ^  0 ^ε, ζ^  0.2254 ^η, θ^ |  |

^α^: Transition probability from day 10 or discharge to month 3

^β^: Transition probability from month 3 to month 6

^γ^: Transition probability from month 6 to month 9

^δ^: Transition probability from month 9 to month 12

^ε^: Transition probability from month 12 to month 15

^ζ^: Transition probability from month 15 to month 18

^η^: Transition probability from month 18 to month 21

^θ^: Transition probability from month 21 to month 24

Transition probabilities were derived by grouping the proportion of patients transitioning from one health state to another one at each time point into a transition matrix. The transition matrices were then converted into hazards and transformed into transition probabilities by applying the appropriate time unit (i.e. 3-month transition probabilities).

Supplemental Table 7 Transition probabilities of subgroup baseline NIHSS 10 to 14.

| Transition from | MLC601 | Placebo | Source |
| --- | --- | --- | --- |
| mRS 0-1 to 2 | 0 ^α^  0.0857 ^β^  0.0107 ^γ, δ^  0.0098 ^ε, ζ^  0.0096 ^η, θ^ | 0 ^α^  0.0526 ^β^  0.0378 ^γ, δ^  0.0194 ^ε, ζ^  0.0187 ^η, θ^ | 11,12 |
| mRS 0-1 to 3 | 0 ^α^  0 ^β^  0.0215 ^γ, δ^  0.0098 ^ε, ζ^  0 ^η, θ^ | 0 ^α^  0 ^β^  0 ^γ, δ^  0.0194 ^ε, ζ^  0 ^η, θ^ |  |
| mRS 0-1 to 4 | 0 ^α^  0 ^β^  0 ^γ, δ^  0 ^ε, ζ^  0.0194 ^η, θ^ | 0 ^α^  0 ^β^  0 ^γ, δ^  0 ^ε, ζ^  0 ^η, θ^ |  |
| mRS 0-1 to 5 | 0 ^α^  0 ^β^  0 ^γ, δ^  0 ^ε, ζ^  0 ^η, θ^ | 0 ^α^  0 ^β^  0 ^γ, δ^  0 ^ε, ζ^  0 ^η, θ^ |  |
| mRS 0-1 to 6 | 0 ^α^  0 ^β^  0 ^γ, δ^  0 ^ε, ζ^  0 ^η, θ^ | 0 ^α^  0 ^β^  0 ^γ, δ^  0 ^ε, ζ^  0 ^η, θ^ |  |
| mRS 2 to 0-1 | 0.5000 ^α^  0.2941 ^β^  0.1181 ^γ, δ^  0.0513 ^ε, ζ^  0.0187 ^η, θ^ | 0.4615 ^α^  0.2286 ^β^  0.0157 ^γ, δ^  0.0742 ^ε, ζ^  0.0298 ^η, θ^ |  |
| mRS 2 to 3 | 0 ^α^  0.0882 ^β^  0.0140 ^γ, δ^  0.0513 ^ε, ζ^  0.0187 ^η, θ^ | 0 ^α^  0.1714 ^β^  0.0481 ^γ, δ^  0.0144 ^ε, ζ^  0.0148 ^η, θ^ |  |
| mRS 2 to 4 | 0 ^α^  0 ^β^  0.0282 ^γ, δ^  0 ^ε, ζ^  0.0187 ^η, θ^ | 0.0769 ^α^  0.0286 ^β^  0 ^γ, δ^  0.0144 ^ε, ζ^  0 ^η, θ^ |  |
| mRS 2 to 5 | 0 ^α^  0 ^β^  0 ^γ, δ^  0 ^ε, ζ^  0 ^η, θ^ | 0 ^α^  0 ^β^  0 ^γ, δ^  0.0144 ^ε, ζ^  0.0148 ^η, θ^ |  |
| mRS 2 to 6 | 0 ^α^  0 ^β^  0 ^γ, δ^  0 ^ε, ζ^  0 ^η, θ^ | 0 ^α^  0 ^β^  0.0157 ^γ, δ^  0 ^ε, ζ^  0 ^η, θ^ |  |
| mRS 3 to 0-1 | 0.4359 ^α^  0.0645 ^β^  0 ^γ, δ^  0.0180 ^ε, ζ^  0 ^η, θ^ | 0.2759 ^α^  0 ^β^  0 ^γ, δ^  0 ^ε, ζ^  0.0132 ^η, θ^ |  |
| mRS 3 to 2 | 0.3333 ^α^  0.2258 ^β^  0.0715 ^γ, δ^  0.0364 ^ε, ζ^  0.0551 ^η, θ^ | 0.4138 ^α^  0.1951 ^β^  0.0675 ^γ, δ^  0.0707 ^ε, ζ^  0.0132 ^η, θ^ |  |
| mRS 3 to 4 | 0.0256 ^α^  0.1290 ^β^  0 ^γ, δ^  0.0551 ^ε, ζ^  0 ^η, θ^ | 0.0345 ^α^  0.0488 ^β^  0.0109 ^γ, δ^  0.0114 ^ε, ζ^  0 ^η, θ^ |  |
| mRS 3 to 5 | 0.0256 ^α^  0 ^β^  0 ^γ, δ^  0 ^ε, ζ^  0 ^η, θ^ | 0.0345 ^α^  0 ^β^  0 ^γ, δ^  0 ^ε, ζ^  0 ^η, θ^ |  |
| mRS 3 to 6 | 0 ^α^  0 ^β^  0 ^γ, δ^  0 ^ε, ζ^  0 ^η, θ^ | 0 ^α^  0 ^β^  0.0109 ^γ, δ^  0 ^ε, ζ^  0.0132 ^η, θ^ |  |
| mRS 4 to 0-1 | 0.1980 ^α^  0.0370 ^β^  0 ^γ, δ^  0 ^ε, ζ^  0 ^η, θ^ | 0.1136 ^α^  0.0588 ^β^  0 ^γ, δ^  0 ^ε, ζ^  0 ^η, θ^ |  |
| mRS 4 to 2 | 0.2178 ^α^  0.0741 ^β^  0 ^γ, δ^  0.0267 ^ε, ζ^  0 ^η, θ^ | 0.2727 ^α^  0.1176 ^β^  0 ^γ, δ^  0 ^ε, ζ^  0 ^η, θ^ |  |
| mRS 4 to 3 | 0.3069 ^α^  0.2222 ^β^  0.0267 ^γ, δ^  0.0267 ^ε, ζ^  0 ^η, θ^ | 0.4205 ^α^  0.2941 ^β^  0.1230 ^γ, δ^  0 ^ε, ζ^  0.0465 ^η, θ^ |  |
| mRS 4 to 5 | 0.0099 ^α^  0.1111 ^β^  0.0267 ^γ, δ^  0.0267 ^ε, ζ^  0 ^η, θ^ | 0 ^α^  0 ^β^  0 ^γ, δ^  0 ^ε, ζ^  0 ^η, θ^ |  |
| mRS 4 to 6 | 0 ^α^  0 ^β^  0 ^γ, δ^  0 ^ε, ζ^  0.0267 ^η, θ^ | 0 ^α^  0 ^β^  0.0109 ^γ, δ^  0.0513 ^ε, ζ^  0 ^η, θ^ |  |
| mRS 5 to 0-1 | 0 ^α^  0 ^β^  0 ^γ, δ^  0 ^ε, ζ^  0 ^η, θ^ | 0 ^α^  0 ^β^  0 ^γ, δ^  0 ^ε, ζ^  0 ^η, θ^ |  |
| mRS 5 to 2 | 0 ^α^  0 ^β^  0 ^γ, δ^  0 ^ε, ζ^  0 ^η, θ^ | 0 ^α^  0 ^β^  0 ^γ, δ^  0 ^ε, ζ^  0 ^η, θ^ |  |
| mRS 5 to 3 | 0.1667 ^α^  0 ^β^  0 ^γ, δ^  0.1340 ^ε, ζ^  0 ^η, θ^ | 0.2500 ^α^  0.3333 ^β^  0 ^γ, δ^  0 ^ε, ζ^  0 ^η, θ^ |  |
| mRS 5 to 4 | 0.8333 ^α^  0 ^β^  0 ^γ, δ^  0 ^ε, ζ^  0.1340 ^η, θ^ | 0.3750 ^α^  0 ^β^  0 ^γ, δ^  0 ^ε, ζ^  0 ^η, θ^ |  |
| mRS 5 to 6 | 0 ^α^  0 ^β^  0.2254 ^γ, δ^  0 ^ε, ζ^  0 ^η, θ^ | 0 ^α^  0.3333 ^β^  0 ^γ, δ^  0 ^ε, ζ^  0 ^η, θ^ |  |

NIHSS: NIH stroke scale

^α^: Transition probability from day 10 or discharge to month 3

^β^: Transition probability from month 3 to month 6

^γ^: Transition probability from month 6 to month 9

^δ^: Transition probability from month 9 to month 12

^ε^: Transition probability from month 12 to month 15

^ζ^: Transition probability from month 15 to month 18

^η^: Transition probability from month 18 to month 21

^θ^: Transition probability from month 21 to month 24

Transition probabilities were derived by grouping the proportion of patients transitioning from one health state to another one at each time point into a transition matrix. The transition matrices were then converted into hazards and transformed into transition probabilities by applying the appropriate time unit (ie. 3-month transition probabilities).

Supplemental Table 8 Transition probabilities of subgroup stroke onset to first dose greater than 48 hours.

| Transition from | MLC601 | Placebo | Source |
| --- | --- | --- | --- |
| mRS 0-1 to 2 | 0 ^α^  0.1053 ^β^  0.0114 ^γ, δ^  0 ^ε, ζ^  0 ^η, θ^ | 0 ^α^  0 ^β^  0.0392 ^γ, δ^  0.0202 ^ε, ζ^  0.0180 ^η, θ^ | 11,12 |
| mRS 0-1 to 3 | 0 ^α^  0.0526 ^β^  0.0230 ^γ, δ^  0.0117 ^ε, ζ^  0.0114 ^η, θ^ | 0 ^α^  0.0526 ^β^  0 ^γ, δ^  0.0202 ^ε, ζ^  0 ^η, θ^ |  |
| mRS 0-1 to 4 | 0 ^α^  0 ^β^  0 ^γ, δ^  0 ^ε, ζ^  0.0114 ^η, θ^ | 0 ^α^  0 ^β^  0 ^γ, δ^  0 ^ε, ζ^  0 ^η, θ^ |  |
| mRS 0-1 to 5 | 0 ^α^  0 ^β^  0.0114 ^γ, δ^  0 ^ε, ζ^  0 ^η, θ^ | 0 ^α^  0.0526 ^β^  0 ^γ, δ^  0 ^ε, ζ^  0 ^η, θ^ |  |
| mRS 0-1 to 6 | 0 ^α^  0 ^β^  0 ^γ, δ^  0 ^ε, ζ^  0 ^η, θ^ | 0 ^α^  0 ^β^  0 ^γ, δ^  0 ^ε, ζ^  0 ^η, θ^ |  |
| mRS 2 to 0-1 | 0.4667 ^α^  0.3030 ^β^  0.0626 ^γ, δ^  0.0513 ^ε, ζ^  0.0211 ^η, θ^ | 0.5333 ^α^  0.2581 ^β^  0.0168 ^γ, δ^  0.0897 ^ε, ζ^  0.0163 ^η, θ^ |  |
| mRS 2 to 3 | 0 ^α^  0.0909 ^β^  0.0153 ^γ, δ^  0.0513 ^ε, ζ^  0 ^η, θ^ | 0.1333 ^α^  0.1613 ^β^  0.0339 ^γ, δ^  0.0438 ^ε, ζ^  0.0163 ^η, θ^ |  |
| mRS 2 to 4 | 0 ^α^  0 ^β^  0.0153 ^γ, δ^  0 ^ε, ζ^  0.0211 ^η, θ^ | 0 ^α^  0.0323 ^β^  0 ^γ, δ^  0.0144 ^ε, ζ^  0.0163 ^η, θ^ |  |
| mRS 2 to 5 | 0 ^α^  0 ^β^  0 ^γ, δ^  0 ^ε, ζ^  0 ^η, θ^ | 0 ^α^  0 ^β^  0 ^γ, δ^  0 ^ε, ζ^  0 ^η, θ^ |  |
| mRS 2 to 6 | 0 ^α^  0 ^β^  0.0153 ^γ, δ^  0 ^ε, ζ^  0 ^η, θ^ | 0 ^α^  0 ^β^  0.0168 ^γ, δ^  0 ^ε, ζ^  0 ^η, θ^ |  |
| mRS 3 to 0-1 | 0.4651 ^α^  0 ^β^  0 ^γ, δ^  0.0230 ^ε, ζ^  0.0211 ^η, θ^ | 0.2500 ^α^  0 ^β^  0 ^γ, δ^  0 ^ε, ζ^  0.0136 ^η, θ^ |  |
| mRS 3 to 2 | 0.3953 ^α^  0.2632 ^β^  0.0707 ^γ, δ^  0.0230 ^ε, ζ^  0.0426 ^η, θ^ | 0.3750 ^α^  0.2791 ^β^  0.0850 ^γ, δ^  0.0646 ^ε, ζ^  0.0414 ^η, θ^ |  |
| mRS 3 to 4 | 0.0233 ^α^  0.0526 ^β^  0 ^γ, δ^  0.0465 ^ε, ζ^  0.0211 ^η, θ^ | 0.0625 ^α^  0.0698 ^β^  0.0117 ^γ, δ^  0 ^ε, ζ^  0.0136 ^η, θ^ |  |
| mRS 3 to 5 | 0 ^α^  0 ^β^  0 ^γ, δ^  0 ^ε, ζ^  0 ^η, θ^ | 0.0312 ^α^  0 ^β^  0 ^γ, δ^  0 ^ε, ζ^  0 ^η, θ^ |  |
| mRS 3 to 6 | 0 ^α^  0 ^β^  0 ^γ, δ^  0 ^ε, ζ^  0 ^η, θ^ | 0 ^α^  0 ^β^  0 ^γ, δ^  0.0126 ^ε, ζ^  0.0136 ^η, θ^ |  |
| mRS 4 to 0-1 | 0.1912 ^α^  0 ^β^  0 ^γ, δ^  0 ^ε, ζ^  0 ^η, θ^ | 0.0741 ^α^  0.0625 ^β^  0 ^γ, δ^  0 ^ε, ζ^  0 ^η, θ^ |  |
| mRS 4 to 2 | 0.2059 ^α^  0.1111 ^β^  0 ^γ, δ^  0 ^ε, ζ^  0 ^η, θ^ | 0.2963 ^α^  0.0625 ^β^  0 ^γ, δ^  0 ^ε, ζ^  0.0572 ^η, θ^ |  |
| mRS 4 to 3 | 0.3235 ^α^  0.1667 ^β^  0.0364 ^γ, δ^  0.0392 ^ε, ζ^  0.0364 ^η, θ^ | 0.4321 ^α^  0.3125 ^β^  0.1340 ^γ, δ^  0 ^ε, ζ^  0.0572 ^η, θ^ |  |
| mRS 4 to 5 | 0 ^α^  0 ^β^  0.0364 ^γ, δ^  0 ^ε, ζ^  0 ^η, θ^ | 0 ^α^  0.0625 ^β^  0.0871 ^γ, δ^  0 ^ε, ζ^  0 ^η, θ^ |  |
| mRS 4 to 6 | 0 ^α^  0 ^β^  0 ^γ, δ^  0 ^ε, ζ^  0.0364 ^η, θ^ | 0 ^α^  0 ^β^  0 ^γ, δ^  0.0646 ^ε, ζ^  0 ^η, θ^ |  |
| mRS 5 to 0-1 | 0 ^α^  0 ^β^  0 ^γ, δ^  0 ^ε, ζ^  0 ^η, θ^ | 0 ^α^  0 ^β^  0 ^γ, δ^  0 ^ε, ζ^  0 ^η, θ^ |  |
| mRS 5 to 2 | 0 ^α^  0 ^β^  0 ^γ, δ^  0 ^ε, ζ^  0 ^η, θ^ | 0 ^α^  0 ^β^  0 ^γ, δ^  0 ^ε, ζ^  0 ^η, θ^ |  |
| mRS 5 to 3 | 0.1667 ^α^  0 ^β^  0 ^γ, δ^  0.2929 ^ε, ζ^  0 ^η, θ^ | 0.3333 ^α^  0.5000 ^β^  0 ^γ, δ^  0 ^ε, ζ^  0 ^η, θ^ |  |
| mRS 5 to 4 | 0.8333 ^α^  0 ^β^  0 ^γ, δ^  0 ^ε, ζ^  0 ^η, θ^ | 0.5000 ^α^  0 ^β^  0 ^γ, δ^  0.1056 ^ε, ζ^  0 ^η, θ^ |  |
| mRS 5 to 6 | 0 ^α^  0 ^β^  0 ^γ, δ^  0.2929 ^ε, ζ^  0 ^η, θ^ | 0 ^α^  0 ^β^  0 ^γ, δ^  0 ^ε, ζ^  0.1835 ^η, θ^ |  |

^α^: Transition probability from day 10 or discharge to month 3

^β^: Transition probability from month 3 to month 6

^γ^: Transition probability from month 6 to month 9

^δ^: Transition probability from month 9 to month 12

^ε^: Transition probability from month 12 to month 15

^ζ^: Transition probability from month 15 to month 18

^η^: Transition probability from month 18 to month 21

^θ^: Transition probability from month 21 to month 24

Transition probabilities were derived by grouping the proportion of patients transitioning from one health state to another one at each time point into a transition matrix. The transition matrices were then converted into hazards and transformed into transition probabilities by applying the appropriate time unit (i.e. 3-month transition probabilities).

Supplemental Table 9 Transition probabilities of subgroup rehabilitation during first 3 months.

| Transition from | MLC601 | Placebo | Source |
| --- | --- | --- | --- |
| mRS 0-1 to 2 | 0 ^α^  0.0690 ^β^  0.0126 ^γ, δ^  0 ^ε, ζ^  0.0120 ^η, θ^ | 0 ^α^  0 ^β^  0.0488 ^γ, δ^  0.0241 ^ε, ζ^  0.2020 ^η, θ^ | 11,12 |
| mRS 0-1 to 3 | 0 ^α^  0 ^β^  0 ^γ, δ^  0.0123 ^ε, ζ^  0.0120 ^η, θ^ | 0 ^α^  0.0556 ^β^  0 ^γ, δ^  0 ^ε, ζ^  0 ^η, θ^ |  |
| mRS 0-1 to 4 | 0 ^α^  0 ^β^  0 ^γ, δ^  0 ^ε, ζ^  0.0120 ^η, θ^ | 0 ^α^  0.0556 ^β^  0 ^γ, δ^  0 ^ε, ζ^  0 ^η, θ^ |  |
| mRS 0-1 to 5 | 0 ^α^  0 ^β^  0.0126 ^γ, δ^  0 ^ε, ζ^  0 ^η, θ^ | 0 ^α^  0.0556 ^β^  0 ^γ, δ^  0 ^ε, ζ^  0 ^η, θ^ |  |
| mRS 0-1 to 6 | 0 ^α^  0 ^β^  0 ^γ, δ^  0 ^ε, ζ^  0 ^η, θ^ | 0 ^α^  0 ^β^  0 ^γ, δ^  0 ^ε, ζ^  0 ^η, θ^ |  |
| mRS 2 to 0-1 | 0.7778 ^α^  0.4138 ^β^  0.0780 ^γ, δ^  0.0742 ^ε, ζ^  0.1115 ^η, θ^ | 0.5625 ^α^  0.1875 ^β^  0.0328 ^γ, δ^  0.1124 ^ε, ζ^  0.0339 ^η, θ^ |  |
| mRS 2 to 3 | 0.1111 ^α^  0.1724 ^β^  0.0253 ^γ, δ^  0.0241 ^ε, ζ^  0.0541 ^η, θ^ | 0.0625 ^α^  0.1875 ^β^  0.0667 ^γ, δ^  0.0308 ^ε, ζ^  0.0168 ^η, θ^ |  |
| mRS 2 to 4 | 0 ^α^  0 ^β^  0 ^γ, δ^  0 ^ε, ζ^  0 ^η, θ^ | 0 ^α^  0.0625 ^β^  0 ^γ, δ^  0 ^ε, ζ^  0.0168 ^η, θ^ |  |
| mRS 2 to 5 | 0 ^α^  0 ^β^  0 ^γ, δ^  0 ^ε, ζ^  0 ^η, θ^ | 0 ^α^  0 ^β^  0 ^γ, δ^  0.0153 ^ε, ζ^  0 ^η, θ^ |  |
| mRS 2 to 6 | 0 ^α^  0 ^β^  0 ^γ, δ^  0 ^ε, ζ^  0 ^η, θ^ | 0 ^α^  0 ^β^  0.0163 ^γ, δ^  0.0082 ^ε, ζ^  0 ^η, θ^ |  |
| mRS 3 to 0-1 | 0.4474 ^α^  0.0417 ^β^  0.0211 ^γ, δ^  0 ^ε, ζ^  0 ^η, θ^ | 0.3030 ^α^  0 ^β^  0 ^γ, δ^  0 ^ε, ζ^  0 ^η, θ^ |  |
| mRS 3 to 2 | 0.3158 ^α^  0.2500 ^β^  0.0871 ^γ, δ^  0.0253 ^ε, ζ^  0.0823 ^η, θ^ | 0.4242 ^α^  0.3125 ^β^  0.1089 ^γ, δ^  0.1094 ^ε, ζ^  0.0619 ^η, θ^ |  |
| mRS 3 to 4 | 0.0526 ^α^  0.1667 ^β^  0 ^γ, δ^  0.0780 ^ε, ζ^  0 ^η, θ^ | 0.0303 ^α^  0.0625 ^β^  0.0451 ^γ, δ^  0.0174 ^ε, ζ^  0 ^η, θ^ |  |
| mRS 3 to 5 | 0 ^α^  0 ^β^  0 ^γ, δ^  0 ^ε, ζ^  0 ^η, θ^ | 0.0303 ^α^  0 ^β^  0 ^γ, δ^  0 ^ε, ζ^  0 ^η, θ^ |  |
| mRS 3 to 6 | 0 ^α^  0 ^β^  0 ^γ, δ^  0 ^ε, ζ^  0 ^η, θ^ | 0 ^α^  0 ^β^  0.0148 ^γ, δ^  0 ^ε, ζ^  0 ^η, θ^ |  |
| mRS 4 to 0-1 | 0.1286 ^α^  0 ^β^  0 ^γ, δ^  0 ^ε, ζ^  0 ^η, θ^ | 0.0462 ^α^  0 ^β^  0 ^γ, δ^  0 ^ε, ζ^  0 ^η, θ^ |  |
| mRS 4 to 2 | 0.2571 ^α^  0 ^β^  0 ^γ, δ^  0.0282 ^ε, ζ^  0 ^η, θ^ | 0.2615 ^α^  0.1765 ^β^  0 ^γ, δ^  0 ^ε, ζ^  0.0465 ^η, θ^ |  |
| mRS 4 to 3 | 0.3000 ^α^  0.2727 ^β^  0 ^γ, δ^  0 ^ε, ζ^  0.0282 ^η, θ^ | 0.4154 ^α^  0.3529 ^β^  0.0871 ^γ, δ^  0.0426 ^ε, ζ^  0 ^η, θ^ |  |
| mRS 4 to 5 | 0.0143 ^α^  0.0455 ^β^  0.0267 ^γ, δ^  0.0572 ^ε, ζ^  0 ^η, θ^ | 0 ^α^  0.0588 ^β^  0.0426 ^γ, δ^  0 ^ε, ζ^  0 ^η, θ^ |  |
| mRS 4 to 6 | 0 ^α^  0 ^β^  0 ^γ, δ^  0 ^ε, ζ^  0.0282 ^η, θ^ | 0 ^α^  0 ^β^  0 ^γ, δ^  0.0426 ^ε, ζ^  0 ^η, θ^ |  |
| mRS 5 to 0-1 | 0 ^α^  0 ^β^  0 ^γ, δ^  0 ^ε, ζ^  0 ^η, θ^ | 0 ^α^  0 ^β^  0 ^γ, δ^  0 ^ε, ζ^  0 ^η, θ^ |  |
| mRS 5 to 2 | 0 ^α^  0 ^β^  0 ^γ, δ^  0 ^ε, ζ^  0 ^η, θ^ | 0 ^α^  0 ^β^  0 ^γ, δ^  0 ^ε, ζ^  0 ^η, θ^ |  |
| mRS 5 to 3 | 0.1667 ^α^  0 ^β^  0 ^γ, δ^  0.1835 ^ε, ζ^  0 ^η, θ^ | 0.2857 ^α^  0.5000 ^β^  0 ^γ, δ^  0 ^ε, ζ^  0 ^η, θ^ |  |
| mRS 5 to 4 | 0.8333 ^α^  0 ^β^  0 ^γ, δ^  0 ^ε, ζ^  0.1835 ^η, θ^ | 0.5714 ^α^  0 ^β^  0 ^γ, δ^  0 ^ε, ζ^  0 ^η, θ^ |  |
| mRS 5 to 6 | 0 ^α^  0 ^β^  0.2929 ^γ, δ^  0.1835 ^ε, ζ^  0 ^η, θ^ | 0 ^α^  0 ^β^  0 ^γ, δ^  0 ^ε, ζ^  0.2929 ^η, θ^ |  |

^α^: Transition probability from day 10 or discharge to month 3

^β^: Transition probability from month 3 to month 6

^γ^: Transition probability from month 6 to month 9

^δ^: Transition probability from month 9 to month 12

^ε^: Transition probability from month 12 to month 15

^ζ^: Transition probability from month 15 to month 18

^η^: Transition probability from month 18 to month 21

^θ^: Transition probability from month 21 to month 24

Transition probabilities were derived by grouping the proportion of patients transitioning from one health state to another one at each time point into a transition matrix. The transition matrices were then converted into hazards and transformed into transition probabilities by applying the appropriate time unit (i.e. 3-month transition probabilities).

Supplemental Figure 1 Tornado diagram for subgroup baseline NIHSS 10 to 14.


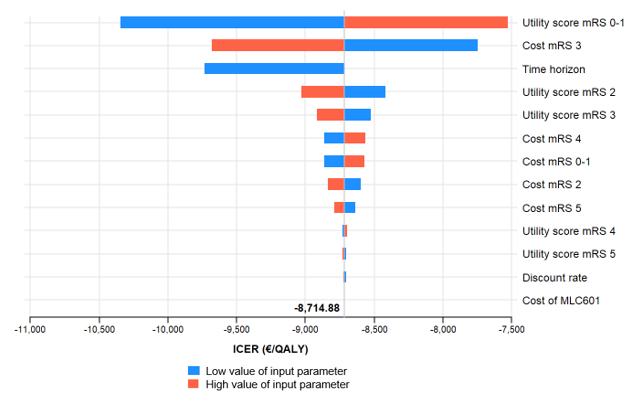


b-NIHSS: baseline NIH Stroke Scale; mRS: modified Rankin Scale; ICER: incremental cost-effectiveness ratio

A Tornado diagram is used to present the results of one-way sensitivity analysis. A list of input parameters was varied one by one, by the lower and higher bound of the mean value of the parameters. The difference of ICERs estimated by varying the extreme values of input parameter was calculated, and arranged from the largest to the lowest difference. The largest difference is shown at the top of the Tornado diagram, and followed by the parameters with smaller difference in ICERs. The Tornado diagram, as suggested by its name, always has a shape of inverted triangle symbolizing the shape of a tornado.

The extreme values (low and high) of each input parameter resulted in negative ICERs signifying the uncertainty around the mean values did not have huge impact to the result.

Supplemental Figure 2 Tornado diagram for subgroup stroke onset to first dose greater than 48 hours.


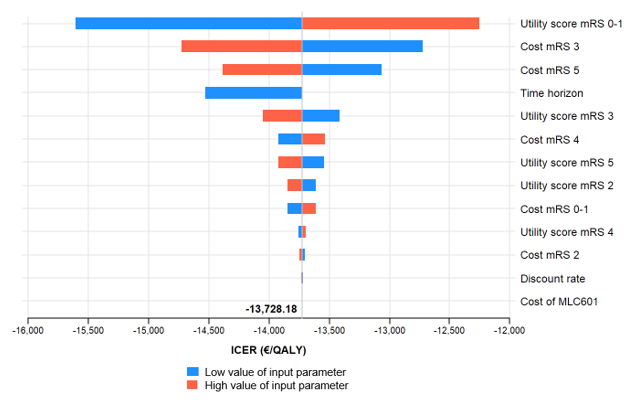


ICER: incremental cost-effectiveness ratio

A Tornado diagram is used to present the results of one-way sensitivity analysis. A list of input parameters was varied one by one, by the lower and higher bound of the mean value of the parameters. The difference of ICERs estimated by varying the extreme values of input parameter was calculated, and arranged from the largest to the lowest difference. The largest difference is shown at the top of the Tornado diagram, and followed by the parameters with smaller difference in ICERs. The Tornado diagram, as suggested by its name, always has a shape of inverted triangle symbolizing the shape of a tornado.

The extreme values (low and high) of each input parameter resulted in negative ICERs signifying the uncertainty around the mean values did not have huge impact to the result.

Supplemental Figure 3 Tornado diagram for subgroup rehabilitation during first 3 months.


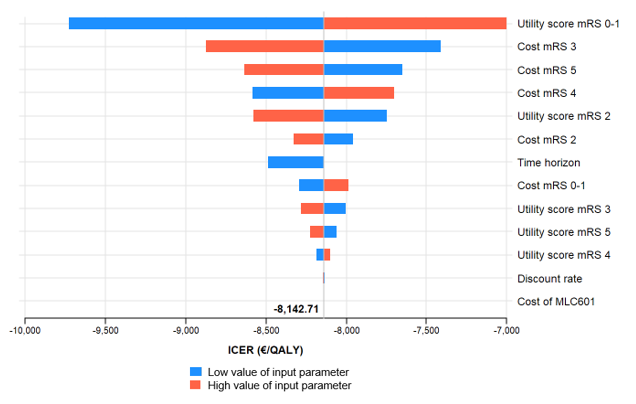


ICER: incremental cost-effectiveness ratio

A Tornado diagram is used to present the results of one-way sensitivity analysis. A list of input parameters was varied one by one, by the lower and higher bound of the mean value of the parameters. The difference of ICERs estimated by varying the extreme values of input parameter was calculated, and arranged from the largest to the lowest difference. The largest difference is shown at the top of the Tornado diagram, and followed by the parameters with smaller difference in ICERs. The Tornado diagram, as suggested by its name, always has a shape of inverted triangle symbolizing the shape of a tornado.

The extreme values (low and high) of each input parameter resulted in negative ICERs signifying the uncertainty around the mean values did not have huge impact to the result.

Supplemental Figure 4 Cost-effectiveness acceptability curve for subgroup baseline NIHSS 10 to 14.


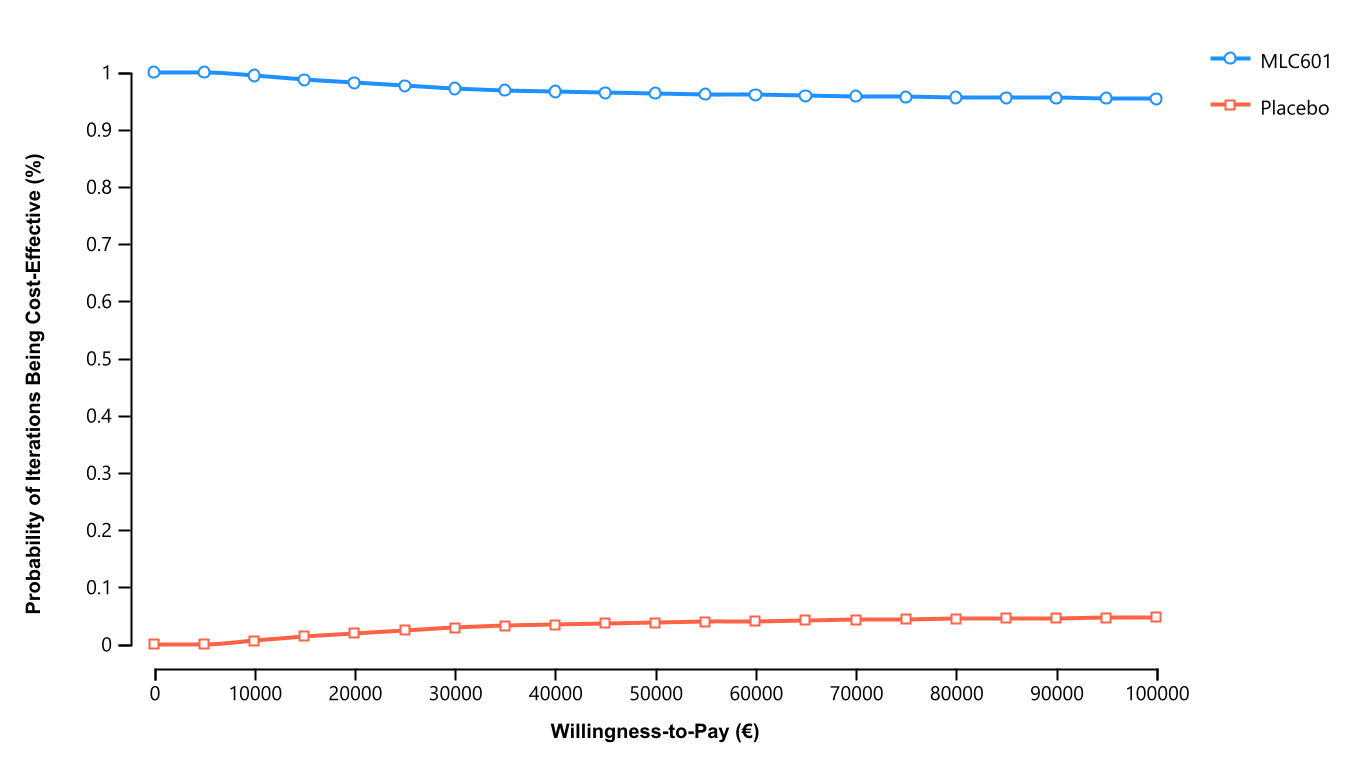


NIHSS: NIH Stroke Scale

The x-axis shows a range of willingness-to-pay (WTP) threshold in Euro and the y-axis represents the probability of the strategy is being cost-effective from Monte-Carlo simulation.

Cost-effectiveness acceptability curve shows the cost-effectiveness results from Monte-Carlo simulation (10,000 iterations) against a range of WTP threshold. The blue line represents MLC601, has always been the preferred strategy, with close to 100% of iterations being cost-effective, across the range of WTP threshold. At a range of WTP threshold from zero to €100,000, MLC601 appears to be cost-effective compared to placebo.

Supplemental Figure 5 Cost-effectiveness acceptability curve for subgroup stroke onset to first dose greater than 48 hours.


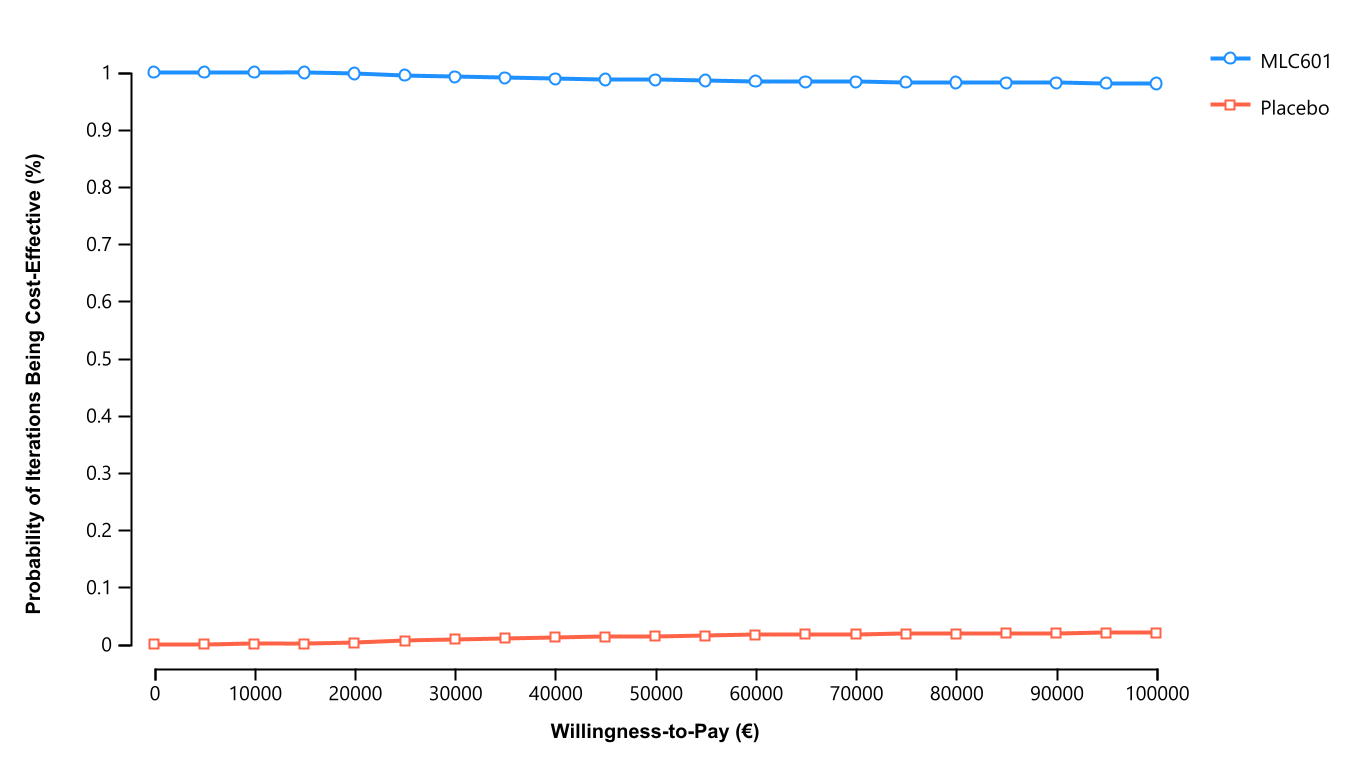


The x-axis shows a range of willingness-to-pay (WTP) threshold in Euro and the y-axis represents the probability of the strategy is being cost-effective from Monte-Carlo simulation.

Cost-effectiveness acceptability curve shows the cost-effectiveness results from Monte-Carlo simulation (10,000 iterations) against a range of WTP threshold. The blue line represents MLC601, has always been the preferred strategy, with close to 100% of iterations being cost-effective, across the range of WTP threshold. At a range of WTP threshold from zero to €100,000, MLC601 appears to be cost-effective compared to placebo.

Supplemental Figure 6 Cost-effectiveness acceptability curve for subgroup rehabilitation during first 3 months.


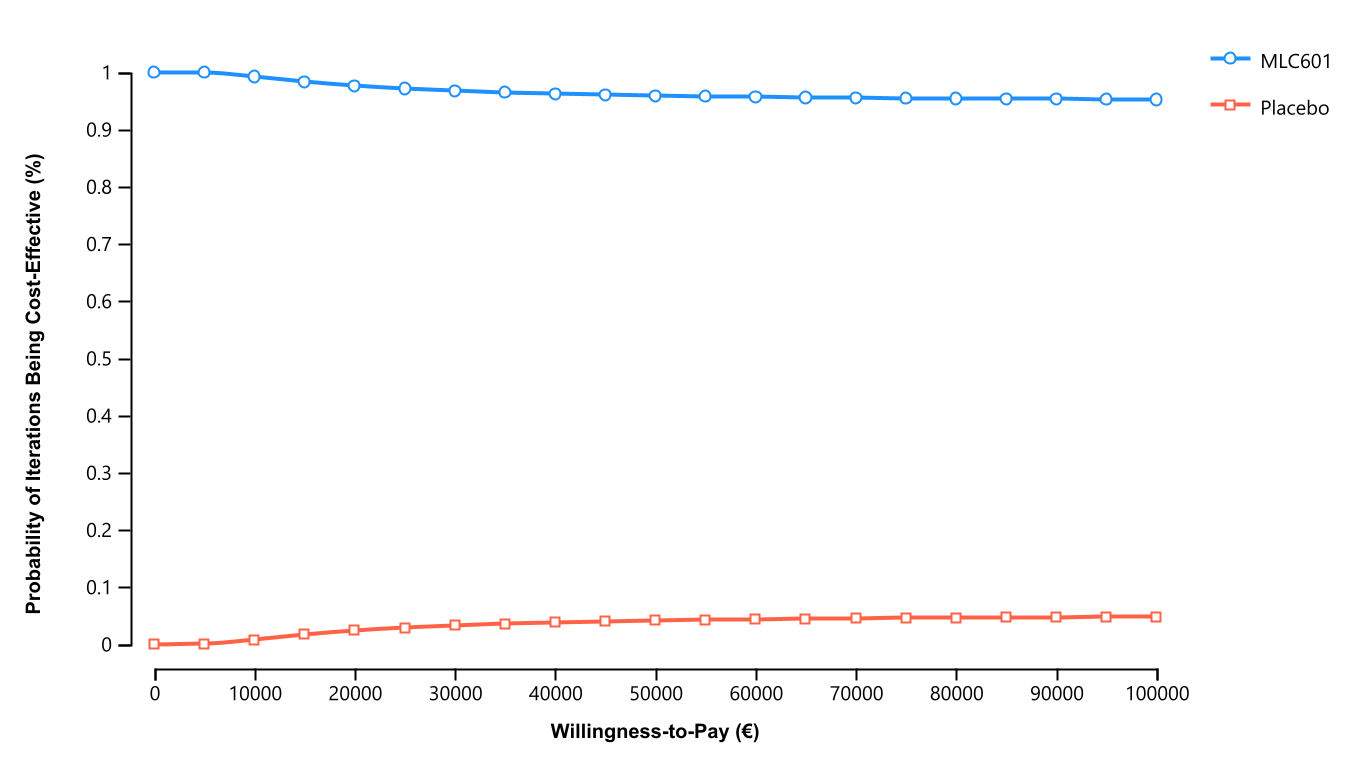


The x-axis shows a range of willingness-to-pay (WTP) threshold in Euro and the y-axis represents the probability of the strategy is being cost-effective from Monte-Carlo simulation.

Cost-effectiveness acceptability curve shows the cost-effectiveness results from Monte-Carlo simulation (10,000 iterations) against a range of WTP threshold. The blue line represents MLC601, has always been the preferred strategy, with close to 100% of iterations being cost-effective, across the range of WTP threshold. At a range of WTP threshold from zero to €100,000, MLC601 appears to be cost-effective compared to placebo.
